# RBM47-Induced Gasdermin A/GSDMA Mediates Mesenchymal–Epithelial Transition and Pyroptosis of Colorectal Cancer Cells

**DOI:** 10.3390/cancers18030504

**Published:** 2026-02-03

**Authors:** Yuyun Du, Matjaz Rokavec, Heiko Hermeking

**Affiliations:** 1 Experimental and Molecular Pathology, Institute of Pathology, Faculty of Medicine, Ludwig-Maximilians-Universität München, Thalkirchner Strasse 36, 80337 Munich, Germany; 2 German Cancer Consortium (DKTK), Partner Site Munich, 80336 Munich, Germany; 3 German Cancer Research Center (DKFZ), 69120 Heidelberg, Germany

**Keywords:** RBM47, GSDMA/Gasdermin A, MET/mesenchymal–epithelial transition, pyroptosis, Oxaliplatin, chemoresistance, colorectal cancer

## Abstract

Down-regulation of the RNA-binding motif protein 47 (RBM47) frequently occurs in colorectal cancer (CRC) and is associated with poor prognosis. However, the downstream effectors of RBM47 have remained unknown. Therefore, we performed a comprehensive RNA-Seq analysis after inactivation or ectopic expression of RBM47. *Gasdermin A*/*GSDMA*, a poorly characterized member of the gasdermin family highly expressed in gastrointestinal epithelium, was identified as the most differentially regulated transcript. RBM47 directly bound to the 3′-untranslated region of *GSDMA* mRNA and stabilized it. Consistently, ectopic *RBM47* increased GSDMA mRNA and protein expression, whereas *RBM47* knockdown had the opposite effect. GSDMA was necessary for the RBM47-induced mesenchymal-to-epithelial transition (MET) and suppression of migration and invasion by RBM47 in CRC cells. Moreover, activation of the RBM47/GSDMA axis triggered pyroptosis, a form of cell death characterized by cell swelling, plasma membrane rupture, and, therefore, immunogenic effects. Both RBM47 and GSDMA enhanced sensitivity to Oxaliplatin through the induction of MET and pyroptosis. Knockdown of GSDMA abolished RBM47-mediated pyroptosis and chemo-sensitization. Analysis of CRC patient cohorts revealed that *RBM47* expression correlates with response to FOLFOX chemotherapy. Therefore, our results establish GSDMA as a critical downstream mediator of RBM47-induced tumor suppression that links epithelial differentiation and pyroptosis to chemotherapy sensitivity. Finally, these findings identify the RBM47/GSDMA axis as a potential predictive biomarker for the response to Oxaliplatin in CRC patients.

## Simple Summary

The RNA-binding motif protein 47 (RBM47) is an RNA-binding protein that is frequently down-regulated in colorectal cancer (CRC). This study addresses a significant gap in understanding how RBM47 mediates tumor suppression, as prior studies had established RBM47 as an antagonist of epithelial-to-mesenchymal transition/EMT and metastasis, but had not pinpointed its critical effectors. Here, we identified *GSDMA* as a key RBM47 target. Binding of RBM47 stabilized the *GSDMA* mRNA, resulting in elevated levels of GSDMA mRNA and protein. GSDMA mediated the RBM47-induced mesenchymal-to-epithelial transition/MET, as well as suppression of migration and invasion. Furthermore, activation of the RBM47/GSDMA regulatory connection triggered pyroptosis, a type of programmed cell death. Notably, the RBM47/GSDMA axis sensitized CRC cells to the chemotherapeutic Oxaliplatin. In CRC patients, elevated RBM47 expression was associated with a better response to FOLFOX chemotherapy, highlighting this pathway as a potential predictive biomarker for responsiveness to chemotherapy.

## 1. Introduction

Colorectal cancer (CRC) is the third most commonly diagnosed cancer worldwide and the second leading cause of cancer death, with more than 1.9 million new cases and over 900,000 deaths estimated in 2022 [1]. Despite advances in early detection and multimodal therapy, the five-year survival rate for patients with metastatic CRC remains below 15% [2]. Oxaliplatin-based chemotherapy regimens, particularly FOLFOX (5-fluorouracil, Leucovorin, and Oxaliplatin), represent the cornerstone of treatment for advanced CRC [3]. However, therapeutic resistance develops in the majority of patients, limiting long-term survival outcomes [4]. Accumulating evidence suggests, that the molecular heterogeneity of CRC contributes to variable treatment responses, underscoring the urgent need to identify predictive biomarkers and elucidate mechanisms underlying chemoresistance [5,6].

RBM47 (RNA-binding motif protein 47) is a RNA-binding protein that regulates post-transcriptional gene expression through multiple mechanisms, including RNA editing, alternative splicing, and modulation of mRNA stability [7]. *RBM47* expression progressively decreases from normal colon epithelium to primary CRC and is further reduced in metastatic lesions [8]. *RBM47* expression is repressed by several epithelial–to-mesenchymal transition (EMT)-related transcription factors and pathways, such as SNAIL, SLUG, IL6/STAT3, and TGFβ/SMAD3 [8,9]. Pathways regulated by RBM47 include WNT and NRF2 signaling: In breast cancer cells, RBM47 stabilizes the *DKK1* mRNA and thereby inhibits Wnt activity [10]. Furthermore, RBM47 was shown to suppress lung cancer growth through the inhibition of NRF2 activity [9]. It has been shown that mice with intestinal epithelial-specific deletion of *RBM47* spontaneously develop polyposis, whereas *RBM47* loss protects against colitis-associated cancer, suggesting that the function of RBM47 in cancer may be context dependent [11]. We recently demonstrated that FOXA1 activates *RBM47* transcription in normal colonic epithelium, whereas silencing of *RBM47* by CpG methylation occurs during tumor progression and is associated with liver metastasis [12]. However, despite these advances in understanding *RBM47* regulation and its association with EMT, the critical downstream effectors mediating RBM47-induced tumor suppression have remained largely unknown.

EMT plays a pivotal role in CRC progression by promoting tumor cell migration, invasion, and metastasis [13,14]. Conversely, mesenchymal-to-epithelial transition (MET) attenuates metastatic potential and sustains a more indolent, tumor-suppressive phenotype [15]. RBM47 maintains epithelial cell identity by antagonizing EMT: Consistently, RBM47 is highly expressed in epithelial-like CRC cells but markedly reduced in mesenchymal-like cells [8,12]. EMT also influences therapeutic resistance, with mesenchymal-like cancer cells exhibiting decreased sensitivity to chemotherapy [16,17,18]. Despite these observations, the specific molecular effectors downstream of RBM47 that mediate MET and contribute to chemotherapy sensitivity have remained unknown [19].

Pyroptosis, a lytic form of inflammatory cell death, is executed by the gasdermin family of pore-forming proteins [20,21]. Proteolytic cleavage and other activation mechanisms release the N-terminal pore-forming domain, which oligomerizes in membranes to form pores, triggering cell lysis and release of pro-inflammatory cytokines and damage-associated molecular patterns (DAMPs) that can activate antitumor immunity [22,23]. Among gasdermin family members, GSDMA is preferentially expressed in differentiated epithelial cells of the gastrointestinal tract and skin, where it contributes to epithelial differentiation and barrier function [24,25,26]. Notably, GSDMA expression is frequently silenced in upper gastrointestinal malignancies, including gastric and esophageal carcinomas, primarily through promoter hypermethylation. Restoration of GSDMA expression inhibits cell proliferation and induces apoptosis in cancer cells, indicating a potentially tumor-suppressive role [24,27,28]. However, the molecular mechanisms governing GSDMA expression beyond transcriptional silencing and the specific roles of GSDMA in regulating epithelial plasticity and response to chemotherapy in CRC have remained largely unexplored.

In this study, we identified GSDMA as a critical downstream effector of RBM47-induced MET and pyroptosis, as well as RBM47-induced suppression of migration and invasion. Using RNA sequencing and functional validation, we demonstrate that RBM47 directly binds to and stabilizes *GSDMA* mRNA, presumably leading to increased GSDMA expression. We show that GSDMA is necessary for RBM47-induced MET and suppression of migration and invasion by RBM47 in CRC cells. Furthermore, the RBM47/GSDMA axis induces pyroptosis, thereby enhancing chemosensitivity to Oxaliplatin. Taken together, our results establish GSDMA as a key mediator of RBM47-regulated epithelial plasticity and pyroptosis. Thereby, RBM47 may influence the response to chemotherapy in CRC patients.

## 2. Results

### 2.1. RNA-Seq Analysis After Knockdown or Ectopic Expression of RBM47

**Figure 1:**
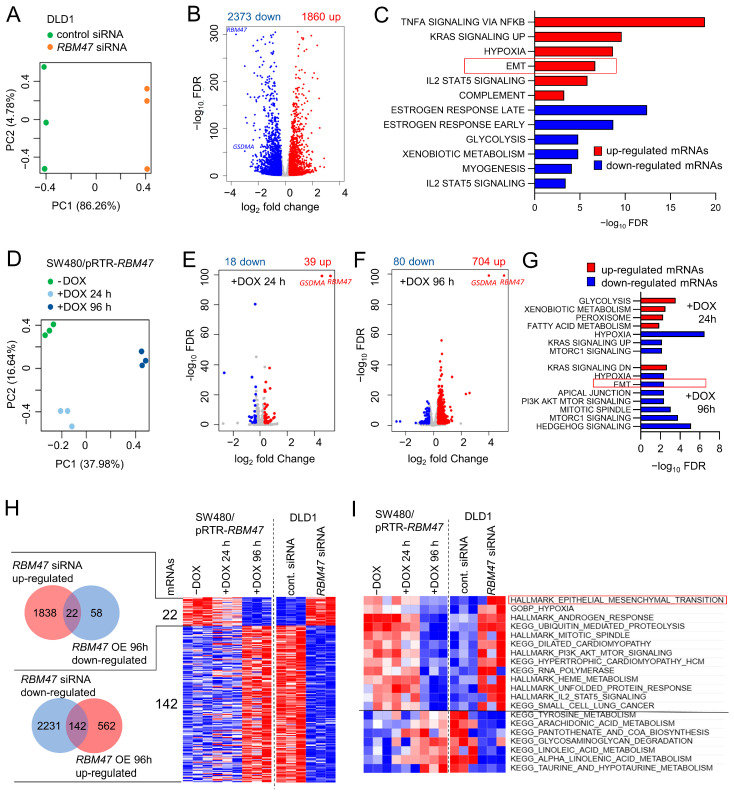
RNA-Seq analysis CRC cell lines after knockdown or ectopic expression of RBM47. (**A**) Principal component analysis of RNA expression in DLD1 cells transfected with control- or RBM47 siRNA. (**B**) Volcano plot showing differential RNA expression between DLD1 cells transfected with control- and RBM47 siRNA with FDR in −log10 scale and fold change in log2 scale. Significantly up- and down-regulated genes (FDR < 0.05 & absolute fold change > 1.25) are highlighted in red and blue, respectively, as indicated. Non-significantly regulated genes are shown in gray. (**C**) Gene Set Enrichment Analysis of mRNAs up- or down-regulated in DLD1 cells transfected with RBM47 siRNA. The most significant MSigDB Hallmark gene sets are shown. (**D**) Principal component analysis of mRNA expression in SW480/pRTR-RBM47 cells treated with vehicle or DOX for 24 and 96 h. (**E**,**F**) Volcano plots showing differential RNA expression between SW480/pRTR-RBM47 cells treated with vehicle or DOX for 24 (**E**) and 96 h (**F**). FDR is shown in −log10 scale, and fold change is presented in log2 scale. Significantly up- and down-regulated genes (FDR < 0.05 & absolute fold change > 1.25) are highlighted in red and blue, respectively, as indicated. Non-significantly regulated genes are shown in gray. (**G**) GSEA of mRNAs up- or down- regulated in SW480/pRTR-RBM47 cells treated with DOX for 24 h and 96 h. The most significant MSigDB Hallmark gene sets are shown. (**H**) Left: Venn diagrams showing the number of differentially expressed mRNAs up-regulated in DLD1 cells after RBM47 siRNA and down-regulated in SW480 cells after ectopic RBM47 expression for 96 h (upper panel) or down-regulated in DLD1 cells after RBM47 siRNA and up-regulated in SW480 cells after ectopic RBM47 expression for 96 h (lower panel). Right: Heat-map of mRNAs up-regulated in DLD1 cells after RBM47 siRNA and down-regulated in SW480 cells after ectopic RBM47 expression for 96 h as well as mRNAs down-regulated in DLD1 cells after RBM47 siRNA and up-regulated in SW480 cells after ectopic RBM47 expression for 96 h. (**I**) Heatmap of GSVA of the indicated pathway activities changes caused by RBM47 overexpression or knockdown.

In order to comprehensively identify downstream effectors of RBM47 function, we performed RNA-Seq analysis of DLD1 CRC cells transfected with either control or *RBM47*-specific siRNAs and of SW480 CRC cells ectopically expressing a doxycycline (DOX)-inducible *RBM47* allele (SW480/pRTR-*RBM47*), which were treated with vehicle or DOX for 24 and 96 h (each condition in three biological replicates). Principal component analysis (PCA) revealed that the majority of differential mRNA regulations after knockdown of *RBM47* were represented by the principal component (PC) 1 (Figure 1A). Consistently, all three replicates of DLD1 cells treated with control siRNAs were well separated from the three replicates of DLD1 cells treated with *RBM47*-specific siRNAs by the PC1. Knockdown of *RBM47* resulted in a significant up-regulation of 1860 mRNAs and down-regulation of 2373 mRNAs (FDR < 0.05 & absolute fold change > 1.25) (Figure 1B). Gene set enrichment analysis (GSEA) showed the most pronounced enrichment within TNF signaling, hypoxia, and EMT-related signatures for up-regulated mRNAs, and estrogen response and glycolysis for down-regulated mRNAs (Figure 1C). After ectopic *RBM47* expression in SW480/pRTR-*RBM47* cells, PCA demonstrated high similarity between the replicates of each treatment condition (vehicle, DOX for 24 h, and DOX for 96 h) (Figure 1D). Differential RNA expression analysis revealed a significant up-regulation of 39 mRNAs and down-regulation of 18 mRNAs (FDR < 0.05 & absolute fold change > 1.25) in cells treated with DOX for 24 h (Figure 1E). DOX treatment for 96 h resulted in 704 significantly up- and 80 down-regulated mRNAs (Figure 1F). In cells treated with DOX for 24 h GSEA showed the most pronounced enrichment within glycolysis and xenobiotic metabolism for up-regulated mRNAs and hypoxia for down-regulated mRNAs (Figure 1G upper panel), whereas in cells treated with DOX for 96 h up-regulated mRNAs were enriched for KRAS signaling and down-regulated mRNAs for Hedgehog signaling, mitotic spindle, and EMT (Figure 1G lower panel). Next, differentially expressed mRNAs shared between cells with ectopic *RBM47* expression and *RBM47* knockdown were determined: 22 mRNAs were up-regulated after *RBM47*-specific siRNA and down-regulated after ectopic *RBM47*, whereas 142 mRNAs were down-regulated after *RBM47*-specific siRNA and up-regulated after ectopic *RBM47* expression (Figure 1H). Gene Set Variation Analysis (GSVA) showed that EMT was among the processes that was decreased after ectopic *RBM47* and increased after *RBM47*-specific siRNA exposure (Figure 1I). On the contrary, multiple amino acid and lipid metabolism-related processes were enhanced after ectopic *RBM47* expression and suppressed after treatment with *RBM47*-specific siRNAs (Figure 1I).

### 2.2. Differential Regulation of GSDMA Expression by RBM47

**Figure 2:**
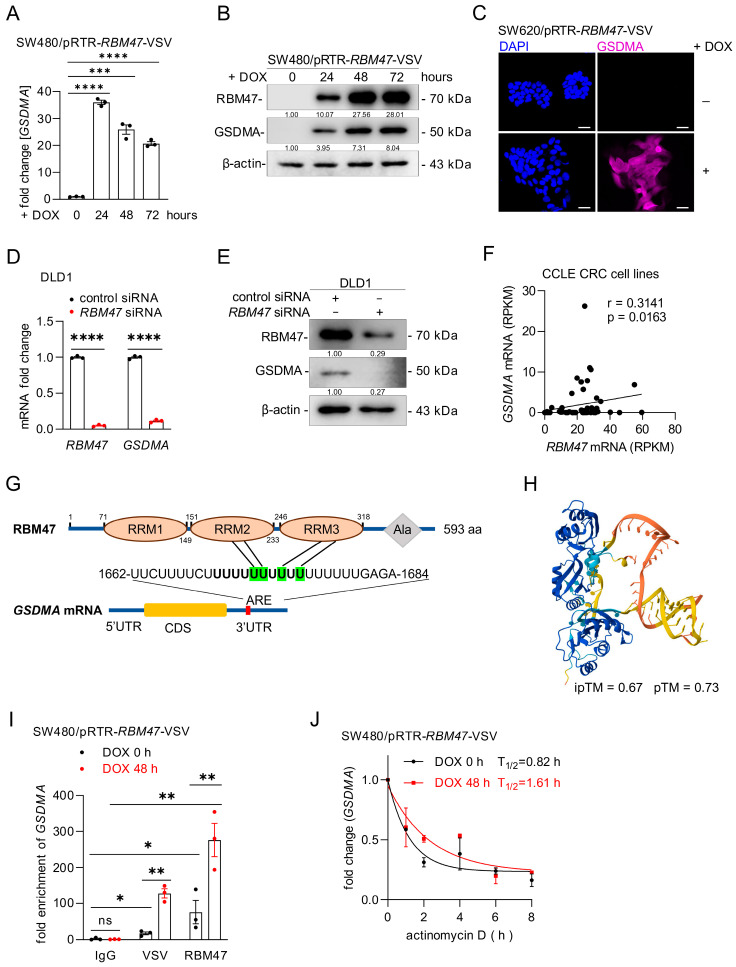
RBM47 induces GSDMA expression by stabilizing GSDMA mRNA. (**A**,**B**) qPCR (**A**) and Western blot (**B**) analyses of RBM47 and GSDMA expression in SW480/pRTR-RBM47-VSV cells treated with or without DOX for indicated time points. Numbers below Western blot bands indicate relative protein levels normalized to β-actin. (**C**) Immunofluorescence staining of GSDMA in SW620/pRTR-RBM47-VSV cells treated with vehicle or DOX for 48 h. Nuclei were counterstained with DAPI. Scale bars, 20 μm. (**D**,**E**) qPCR (**D**) and Western blot (**E**) analyses of RBM47 and GSDMA expression in DLD1 cells 72 h after transfection with control or *RBM47*-specific siRNA. Numbers below bands indicate relative protein levels normalized to β-actin. (**F**) Correlation between *RBM47* and *GSDMA* mRNA expression in human CRC cell lines from the Cancer Cell Line Encyclopedia (CCLE). The Pearson correlation coefficient with two-tailed *p*-value is shown. (**G**) Schematic diagram of RBM47 protein structure showing three RNA recognition motifs (RRM1-3) and alanine-rich region (Ala), and GSDMA mRNA structure with indicated regions. The putative RBM47 binding site (AU-rich element, ARE) within the *GSDMA* 3′-UTR is highlighted. (**H**) Predicted structural model of RBM47-*GSDMA* mRNA interaction generated by AlphaFold3. Interface predicted template modeling score (ipTM) and predicted template modeling score (pTM) are shown. (**I**) RIP-qPCR analysis of *GSDMA* mRNA enrichment in SW480/pRTR-RBM47-VSV cells treated with or without DOX for 48 h. RNA was immunoprecipitated using anti-VSV, anti-RBM47, or control IgG antibodies. Results are expressed as fold enrichment relative to IgG control. (**J**) *GSDMA* mRNA stability assay in SW480/pRTR-RBM47-VSV cells treated with or without DOX for 48 h, followed by actinomycin D treatment for indicated time points. *GSDMA* mRNA half-lives (T_1_/_2_) are indicated. Mean values ± SD (n = 3) are provided. ns, not significant; * *p* < 0.05; ** *p* < 0.01; *** *p* < 0.001; **** *p* < 0.0001.

Among the 142 mRNAs that were down-regulated after *RBM47* knockdown and up-regulated after ectopic *RBM47* expression, *GSDMA* showed the most pronounced fold changes in both experimental conditions (Figure 1B,E,F). GSDMA (Gasdermin A) is a member of the gasdermin family of pore-forming proteins that execute pyroptosis, a form of lytic inflammatory cell death [29]. To validate the RNA-Seq results, we performed qPCR analysis of *GSDMA* expression after treatment of SW480/pRTR-*RBM47*-VSV cells treated with DOX. *GSDMA* mRNA expression was significantly induced in a time-dependent manner, with peak induction at 24 h after DOX treatment (Figure 2A). Also, GSDMA protein expression was induced upon DOX treatment (Figure 2B). Immunofluorescence analysis confirmed the induction of GSDMA protein in SW620/pRTR-*RBM47*-VSV cells following DOX treatment (Figure 2C). Similar results were obtained in SW620 cells, and early time-course experiments (4–18 h) in both SW480 and SW620 cells showed that GSDMA protein was detectable as early as 4–6 h after induction of ectopic *RBM47* (Figure S1A–D). Conversely, siRNA-mediated knockdown of *RBM47* in DLD1 and HCT15 cells resulted in a significant decrease in both *RBM47* and *GSDMA* mRNA and a pronounced reduction in their protein levels (Figure 2D–E and Figure S1E,F). Furthermore, analysis of 53 CRC cell lines from the Cancer Cell Line Encyclopedia (CCLE) [30] revealed a significant positive correlation between *RBM47* and *GSDMA* expression (Figure 2F).

RBM47 contains three RNA recognition motifs (RRMs) and regulates mRNA stability by binding to AU-rich elements (AREs) in the 3′-untranslated region (3′-UTR) of target transcripts [7]. We identified a putative ARE motif in the *GSDMA* 3′-UTR (Figure 2G) and used AlphaFold3 [31] to model the RBM47-*GSDMA* mRNA interaction (Figure 2H). The predicted structure showed high confidence scores (interface predicted template modeling score, ipTM = 0.67; predicted template modeling score, pTM = 0.73), suggesting direct binding. In order to validate this interaction experimentally, RNA immunoprecipitation (RIP) followed by qPCR in SW480/pRTR-*RBM47*-VSV cells treated with or without DOX for 48 h was performed. *GSDMA* mRNA was significantly enriched after immunoprecipitation with VSV- and RBM47-specific antibodies when compared to control IgG, with enrichment being markedly increased upon ectopic *RBM47* expression (Figure 2I). We next assessed whether RBM47 stabilizes *GSDMA* mRNA by treating cells with the transcription inhibitor actinomycin D following RBM47 induction and measuring *GSDMA* mRNA decay. Indeed, ectopic *RBM47* expression extended *GSDMA* mRNA half-life from 0.82 h to 1.61 h in SW480 cells (Figure 2J) and from 1.27 h to 2.59 h in SW620 cells (Figure S1G). Altogether, these results show that RBM47 directly binds to *GSDMA* mRNA and stabilizes the transcript, leading to increased *GSDMA* expression in CRC cells.

### 2.3. Characterization of RBM47 Induced Mesenchymal–Epithelial Transition

**Figure 3:**
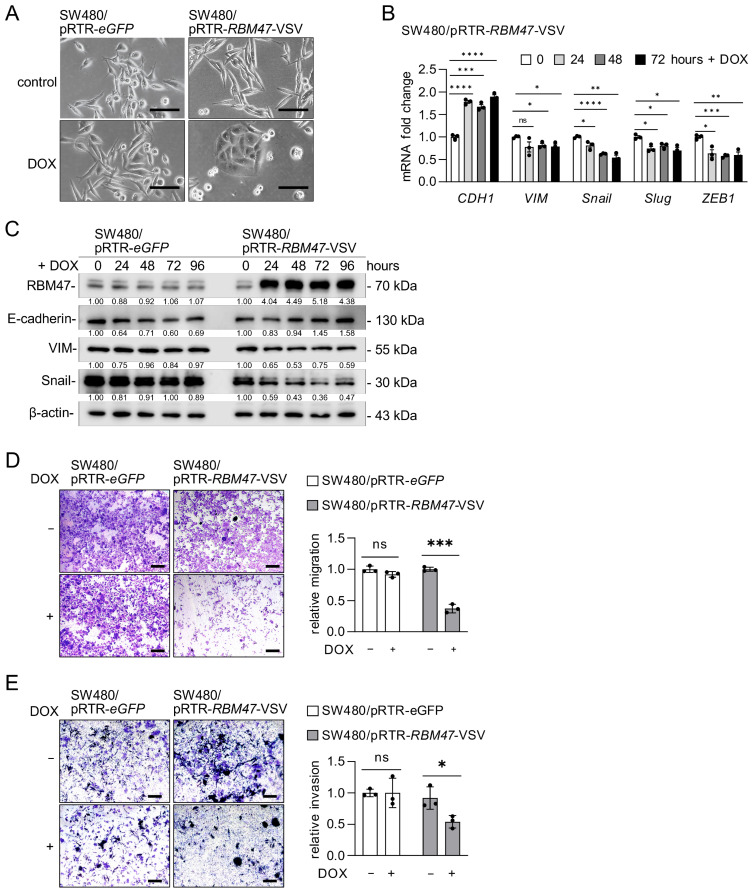
RBM47 induces MET and suppresses migration and invasion in SW480 cells. (**A**) Representative phase-contrast images of SW480/pRTR-eGFP and SW480/pRTR-RBM47-VSV cells treated with vehicle or DOX for 72 h, showing morphological changes from mesenchymal to epithelial phenotype. Scale bars, 50 μm. (**B**) qPCR analysis of EMT-related genes (CDH1, VIM, Snail, Slug, and ZEB1) in SW480/pRTR-RBM47-VSV cells treated with DOX for indicated time points. (**C**) Western blot analysis of RBM47, E-cadherin, VIM, and Snail protein expression in SW480/pRTR-eGFP and SW480/pRTR-RBM47-VSV cells treated with DOX for indicated time points. Numbers below bands indicate relative protein levels normalized to β-actin. (**D**,**E**) Representative images and quantification of Transwell migration (**D**) and invasion (**E**) assays in SW480/pRTR-eGFP and SW480/pRTR-RBM47-VSV cells treated with or without DOX for 48 h. Migrated and invaded cells were stained with crystal violet. Scale bars, 50 μm. Mean values ± SD (n = 3) are provided. ns, not significant; * *p* < 0.05; ** *p* < 0.01; *** *p* < 0.001; **** *p* < 0.0001.

Since the RNA-Seq analysis showed that ectopic RBM47 expression resulted in the down-regulation of EMT-related gene signatures (Figure 1G,I), we investigated whether RBM47 regulates EMT/MET in CRC cells. Ectopic expression of *RBM47* in mesenchymal-like SW480 cells promoted a transition from a mesenchymal to an epithelial morphology, as evidenced by the change from spindle-shaped cells with a scattered growth pattern to tightly packed, cobblestone-like cells (Figure 3A). This morphological change was accompanied by significant alterations in EMT/MET marker expression. qPCR analysis showed that ectopic RBM47 expression induced *CDH1* (E-cadherin) mRNA and suppressed the expression of mesenchymal markers *VIM* (Vimentin), *SNAIL*, *SLUG*, and *ZEB1* in a time-dependent manner (Figure 3B). Consistent with these results, Western blot analysis confirmed that ectopic RBM47 expression induced E-cadherin protein and suppressed VIM and Snail protein levels (Figure 3C). Similar results were observed in SW620 cells, where ectopic RBM47 expression induced morphological changes from mesenchymal to epithelial phenotype, altered EMT/MET marker expression at both mRNA and protein levels, and promoted E-cadherin accumulation at the cell membrane (Figure S2A–D). The effects of DOX on EMT/MET markers observed here were due to ectopic RBM47 expression, as treatment with DOX did not alter the expression of these markers in control cells expressing eGFP (Figure S2E,F).

Since EMT promotes cell migration and invasion, whereas MET suppresses these processes, we examined whether RBM47-induced MET affects the migratory and invasive capacity of CRC cells. Indeed, ectopic expression of *RBM47* significantly suppressed both migration and invasion of SW480 cells compared to control cells, as determined by Transwell assays (Figure 3D,E). Similar results were obtained in SW620 cells, where ectopic RBM47 expression markedly reduced migration and invasion (Figure S2G,H). Taken together, these results demonstrate that RBM47 induces MET and suppresses the migratory and invasive capacity of CRC cells.

### 2.4. RBM47-Induced GSDMA Mediates MET

**Figure 4:**
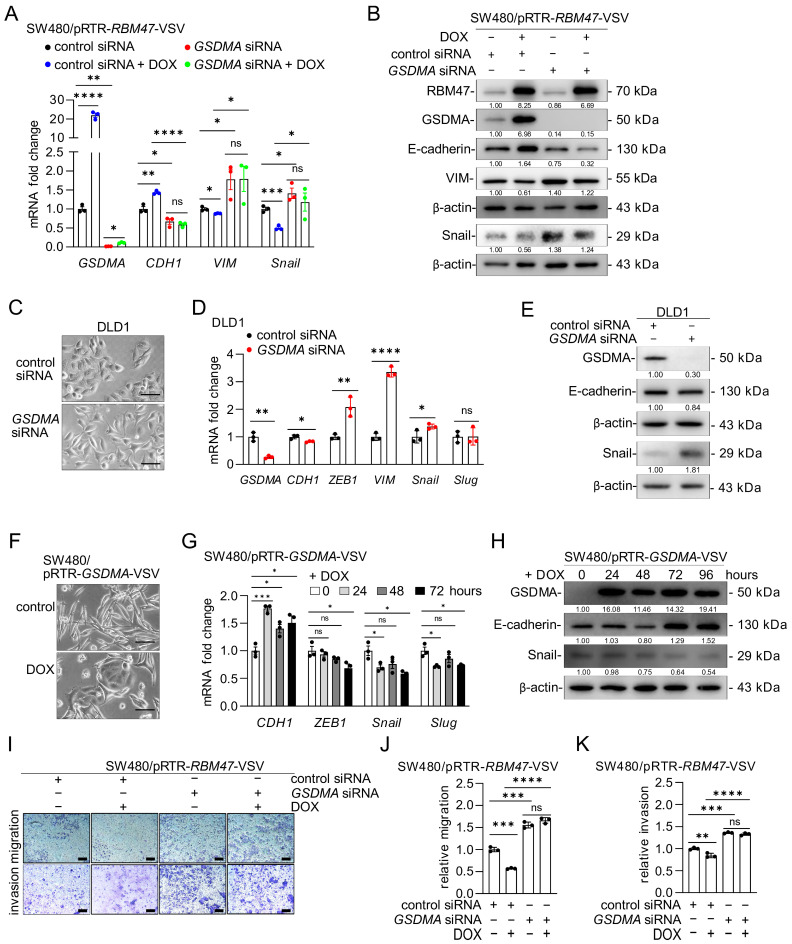
RBM47 induces MET through GSDMA. (**A**,**B**) qPCR (**A**) and Western blot (**B**) analyses of GSDMA and EMT-related genes (CDH1, VIM, Snail) in SW480/pRTR-RBM47-VSV cells transfected with control or GSDMA siRNA and treated with or without DOX for 72 h. Numbers below Western blot bands indicate relative protein levels normalized to β-actin. (**C**) Representative phase-contrast images of DLD1 cells 72 h after transfection with control or GSDMA siRNA. Scale bars, 50 μm. (**D**) qPCR analysis of *GSDMA* and EMT-related mRNAs (*CDH1*, *ZEB1*, *VIM*, *Snail*, *Slug*) in DLD1 cells 72 h after transfection with control or *GSDMA*-specific siRNA. (**E**) Western blot analysis of GSDMA, E-cadherin, and Snail protein expression in DLD1 cells 72 h after transfection with control or *GSDMA*-specific siRNA. Numbers below bands indicate relative protein levels normalized to β-actin. (**F**) Representative phase-contrast images of SW480/pRTR-*GSDMA*-VSV cells treated with vehicle or DOX for 72 h. Scale bars, 50 μm. (**G**,**H**) qPCR (**G**) and Western blot (**H**) analyses of EMT-related mRNAs (*CDH1*, *ZEB1*, *Snail*, *Slug*) and proteins (E-cadherin, Snail) in SW480/pRTR-*GSDMA*-VSV cells treated with DOX for indicated time points. (**I**) Representative images of Transwell migration (upper panel) and invasion (lower panel) assays in SW480/pRTR-*RBM47*-VSV cells transfected with control or *GSDMA*-specific siRNAs and treated with or without DOX for 48 h. Migrated and invaded cells were stained with crystal violet. Scale bars, 50 μm. (**J**,**K**) Quantification of relative migration (**J**) and invasion (**K**) from Transwell assays shown in (**I**). Numbers below Western blot bands indicate relative protein levels normalized to β-actin. Mean values ± SD (n = 3) are provided. ns, not significant; * *p* < 0.05; ** *p* < 0.01; *** *p* < 0.001; **** *p* < 0.0001.

To determine whether the MET-inducing effects of RBM47 are mediated by GSDMA, we knocked down *GSDMA* in SW480/pRTR-*RBM47*-VSV cells and induced ectopic *RBM47* expression by addition of DOX. In cells transfected with control siRNA, ectopic RBM47 expression induced *GSDMA* and *CDH1* mRNA and suppressed *VIM* and *SNAIL* mRNA (Figure 4A). However, siRNA-mediated knockdown of *GSDMA* prevented these RBM47-induced changes in EMT/MET marker expression (Figure 4A). Western blot analysis confirmed that *GSDMA* knockdown blocked RBM47-induced E-cadherin expression and, to a large extent, the suppression of VIM and Snail (Figure 4B). Similar results were obtained in SW620 cells (Figure S3A,B). We next examined the effects of *GSDMA* knockdown in epithelial-like DLD1 and HCT15 cells. siRNA-mediated *GSDMA* depletion induced morphological changes typical for EMT in both cell lines (Figure 4C and Figure S3C), suppressed *CDH1* expression, and induced mesenchymal markers *VIM*, *SNAIL*, *ZEB1*, and *SLUG* (Figure 4D,E and Figure S3D,E). On the other hand, ectopic expression of GSDMA in mesenchymal-like SW480 and SW620 cells promoted morphological changes towards an epithelial phenotype (Figure 4F and Figure S3F), induced *CDH1* expression, and suppressed mesenchymal markers *ZEB1*, *SNAIL*, and *SLUG* at both mRNA and protein levels (Figure 4G,H and Figure S3G–I). These results demonstrate that GSDMA is both necessary and sufficient for MET in CRC cells.

Since RBM47 suppresses migration and invasion through inducing MET, we determined whether GSDMA is required for these effects. In SW480 cells transfected with control siRNA, ectopic RBM47 expression significantly suppressed both migration and invasion (Figure 4I–K). However, *GSDMA* knockdown abolished the inhibitory effects of RBM47 on migration and invasion (Figure 4I–K). Altogether, these results show that RBM47 induces MET and suppresses migration and invasion through the induction of *GSDMA* in CRC cells.

### 2.5. RBM47-Induced GSDMA Suppresses Cell Proliferation and Induces Pyroptosis

**Figure 5:**
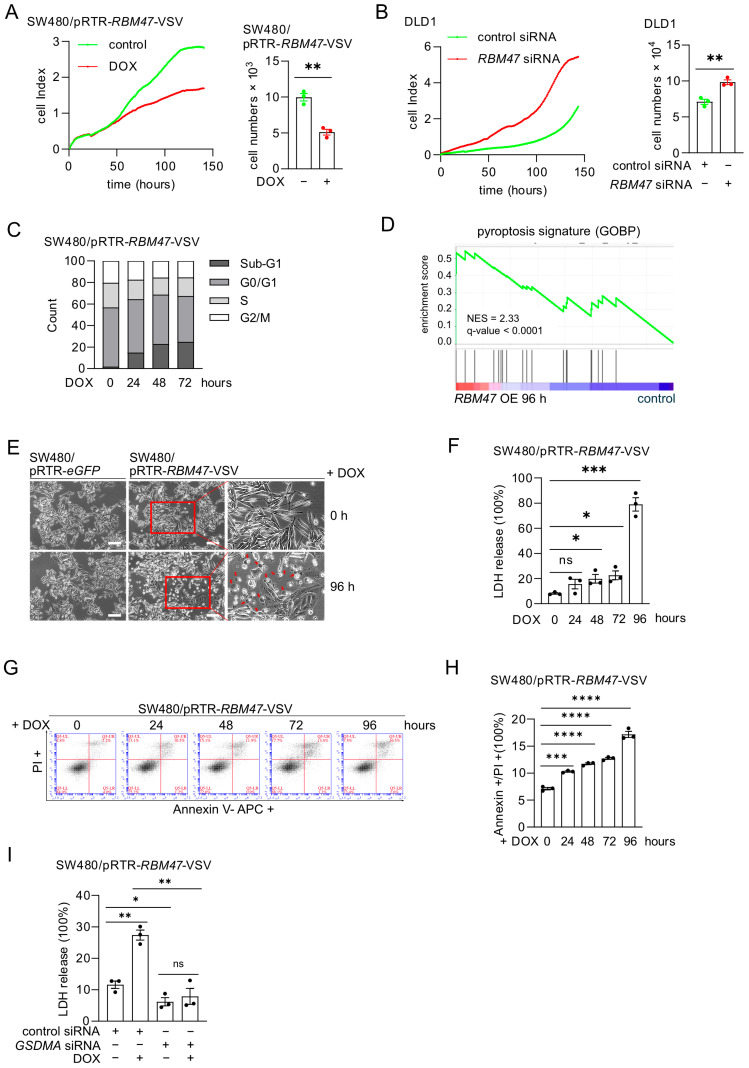
RBM47 suppresses cell proliferation by inducing pyroptosis in CRC cells. (**A**) Cell impedance (left) and cell number quantification (right) of SW480/pRTR-RBM47-VSV cells treated with or without DOX. Cell index was measured by real-time cell analysis. (**B**) Cell impedance (left) and cell number quantification (right) of DLD1 cells transfected with control or *RBM47*-specific siRNA. (**C**) Flow cytometric DNA-content analysis in SW480/pRTR-*RBM47*-VSV cells treated with DOX for indicated time points. Cell cycle phases (Sub-G_1_, G_0_/G_1_, S, and G_2_/M) are indicated. (**D**) Gene Set Enrichment Analysis (GSEA) showing enrichment of the pyroptosis gene signature (GOBP_Pyroptotic_Inflammatory_response (MSigDB)) in SW480/pRTR-*RBM47*-VSV cells treated with doxycycline (DOX) for 96 h compared to vehicle-treated control cells. Normalized enrichment score (NES) and q-value are indicated. (**E**) Representative phase-contrast images of SW480/pRTR-*eGFP* and SW480/pRTR-*RBM47*-VSV cells treated with DOX for 0 and 96 h. Red arrows indicate cells undergoing pyroptosis. Scale bars, 50 μm. (**F**) Quantification of LDH release in SW480/pRTR-*RBM47*-VSV cells treated with DOX for the indicated periods. (**G**,**H**) Flow cytometry analysis (**G**) and quantification (**H**) of Annexin V and PI double-positive cells in SW480/pRTR-*RBM47*-VSV cells treated with DOX for the indicated periods. (**I**) Quantification of LDH release in SW480/pRTR-*RBM47*-VSV cells transfected with control or *GSDMA*-specific siRNA and treated with or without DOX for 96 h. Mean values ± SD (n = 3) are provided. ns, not significant; * *p* < 0.05; ** *p* < 0.01; *** *p* < 0.001; **** *p* < 0.0001.

To investigate the effects of RBM47 on cell proliferation and viability, we monitored cellular impedance as a proxy for cell numbers in SW480/pRTR-*RBM47*-VSV cells treated with DOX. Ectopic expression of RBM47 significantly suppressed cellular impedance (Figure 5A, left panel). Cell counting at the end of the analysis confirmed that these effects were due to a decrease in the cell numbers (Figure 5A, right panel). Conversely, siRNA-mediated knockdown of *RBM47* in DLD1 cells increased the number of cells (Figure 5B and Figure S4A). Similar results were obtained in SW620 and HCT15 cells (Figure S4B–E). Flow cytometry analysis showed that ectopic RBM47 expression in SW480 and SW620 cells increased the number of cells with a sub-G_1_ DNA content (Figure 5C and Figure S4F,G), implying that RBM47 induces cell death. In addition, minor decreases of cells in S- and G_2_/M-phases were observed, which may also contribute to decreased proliferation. GSEA revealed that the GOBP_Pyroptotic_Inflammatory_Response (MSigDB) gene signature was significantly enriched in SW480 cells after ectopic RBM47 expression for 96 h (Figure 5D). Pyroptosis is a form of lytic, inflammatory cell death characterized by cell swelling, plasma membrane rupture, and release of intracellular contents [32]. qPCR analysis showed that ectopic RBM47 expression did not significantly alter the expression of the other gasdermin family members *GSDMB*, *GSDMC*, *GSDMD*, and *GSDME* (Figure S5A,B). Western blot analysis detected full-length GSDMA protein but no N-terminal cleavage product after ectopic RBM47 expression (Figure S5C), indicating that RBM47 induces pyroptosis without canonical GSDMA cleavage.

To determine the type of cell death observed here, we examined morphological and biochemical markers of pyroptotic cell death. Phase-contrast microscopy showed that ectopic expression of RBM47 in SW480 and SW620 cells induced cell swelling and membrane blebbing (Figure 5E and Figure S5D), which are morphological features consistent with pyroptotic cell death. Lactate dehydrogenase (LDH) release, a marker of plasma membrane rupture, was significantly increased in a time-dependent manner after ectopic RBM47 expression in SW480 and SW620 cells (Figure 5F and Figure S5E). In contrast, DOX treatment did not induce LDH release in control cells expressing eGFP (Figure S5F,G). Flow cytometry analysis showed that ectopic RBM47 expression significantly increased the percentage of Annexin V and propidium iodide (PI) double-positive SW480 and SW620 cells (Figure 5G,H and Figure S5H,I), further confirming membrane permeabilization and cell death. To determine whether GSDMA is required for RBM47-induced pyroptosis, we knocked down *GSDMA* in SW480 and SW620 cells expressing ectopic RBM47. *GSDMA* knockdown significantly reduced LDH release induced by RBM47 (Figure 5I and Figure S5J). Taken together, these results demonstrate that RBM47 induces pyroptosis through GSDMA in CRC cells which presumably contributes to reduced cell proliferation.

### 2.6. RBM47-Induced GSDMA Enhances Sensitivity of CRC Cells to Oxaliplatin

**Figure 6:**
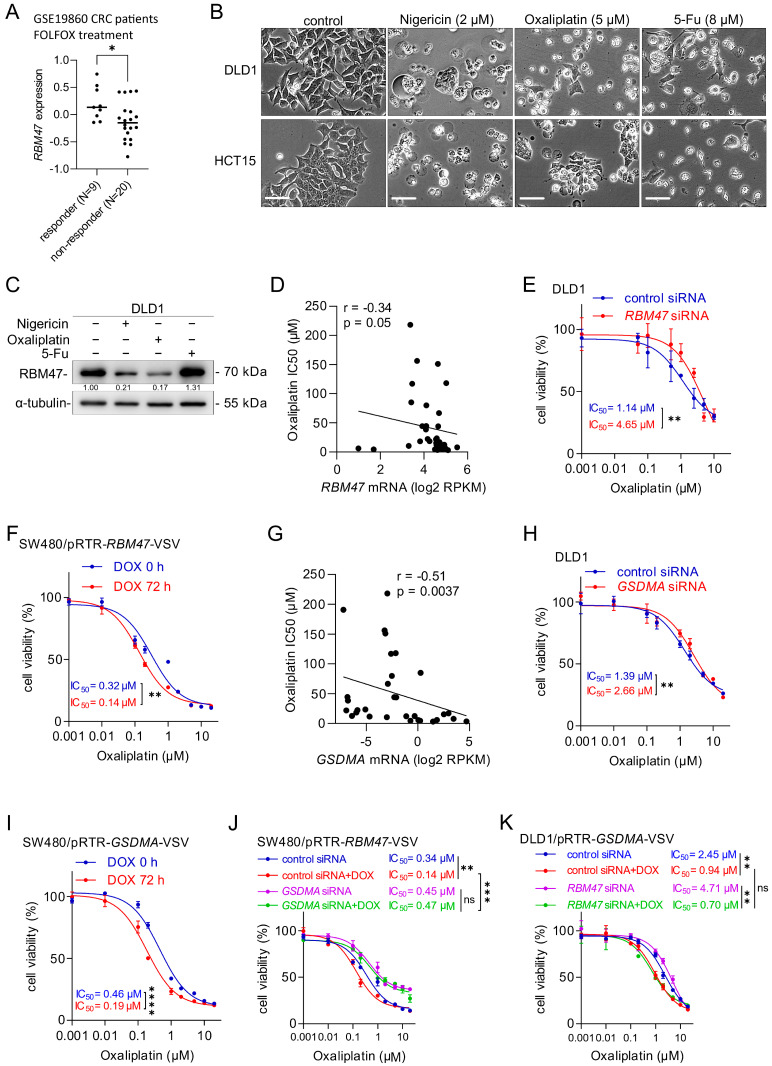
RBM47 enhances chemosensitivity of CRC cells to Oxaliplatin via GSDMA. (**A**) *RBM47* mRNA expression in CRC patients from the GSE19860 cohort who received FOLFOX treatment. Patients were stratified into responders (n = 9) and non-responders (n = 20) based on treatment outcome. (**B**) Representative phase-contrast images of DLD1 and HCT15 cells treated with vehicle (control), Nigericin (2 μM), Oxaliplatin (5 μM), or 5-fluorouracil (5-Fu, 8 μM) for 48 h, showing pyroptosis-specific morphological changes. Scale bars, 50 μm. (**C**) Western blot analysis of RBM47 protein expression in DLD1 cells treated with Nigericin (2 μM), Oxaliplatin (5 μM), or 5-Fu (8 μM) for 48 h. α-tubulin served as a loading control. (**D**) Correlation between *RBM47* mRNA expression and Oxaliplatin IC50 values across 36 human CRC cell lines (data from CCLE and GDSC). The Pearson correlation coefficient (r) with two-tailed *p*-value is shown. (**E**) Cell viability curves of DLD1 cells transfected with control or *RBM47*-specific siRNA and treated with increasing concentrations of Oxaliplatin for 72 h. IC50 values are indicated. (**F**) Cell viability curves of SW480/pRTR-*RBM47*-VSV cells treated with or without doxycycline (DOX) for 72 h, followed by Oxaliplatin treatment at increasing concentrations for 72 h. IC50 values are indicated. (**G**) Correlation between *GSDMA* mRNA expression and Oxaliplatin IC50 values across 36 human CRC cell lines (data from CCLE and GDSC). The Pearson correlation coefficient (r) with two-tailed *p*-value is shown. (**H**) Cell viability curves of DLD1 cells transfected with control or *GSDMA*-specific siRNA and treated with increasing concentrations of Oxaliplatin for 72 h. IC50 values are indicated. (**I**) Cell viability curves of SW480/pRTR-*GSDMA*-VSV cells treated with or without DOX for 72 h, followed by Oxaliplatin treatment at increasing concentrations for 72 h. IC50 values are indicated. (**J**) Cell viability curves of SW480/pRTR-*RBM47*-VSV cells transfected with control or *GSDMA*-specific siRNA, treated with or without DOX, and exposed to increasing concentrations of Oxaliplatin for 72 h. IC50 values are indicated. (**K**) Cell viability curves of DLD1/pRTR-*GSDMA*-VSV cells transfected with control or *RBM47*-specific siRNA, treated with or without DOX, and exposed to increasing concentrations of Oxaliplatin for 72 h. IC50 values are indicated. Mean values ± SD (n = 3) are provided. ns, not significant; * *p* < 0.05; ** *p* < 0.01; *** *p* < 0.001; **** *p* < 0.0001.

Given that pyroptotic cell death sensitizes cancer cells to chemotherapy [33,34], we investigated whether RBM47 and GSDMA affect the response of CRC cells to chemotherapeutic agents. Analysis of the GSE19860 cohort showed that *RBM47* expression was significantly higher in CRC patients who responded to FOLFOX chemotherapy compared to non-responders (Figure 6A). Given the available sample size (N = 29), the achievable power of detecting this effect at α = 0.05 was approximately 78%. To examine whether chemotherapeutic agents induce pyroptosis, we treated DLD1 and HCT15 cells with Nigericin (a known inducer of pyroptosis), Oxaliplatin, or 5-Fluorouracil (5-FU). Both Nigericin and Oxaliplatin, but not 5-FU, induced cell swelling and membrane blebbing characteristic of pyroptotic cell death (Figure 6B). Western blot analysis showed that Oxaliplatin-treated DLD1 and HCT15 cells displayed reduced RBM47 protein levels (Figure 6C and Figure S6A). This effect may be due to the selection of cells expressing low levels of RBM47 protein, since these are presumably more resistant to Oxaliplatin. Indeed, analysis of 36 CRC cell lines from the Cancer Cell Line Encyclopedia (CCLE) and Genomics of Drug Sensitivity in Cancer (GDSC) database [30,35] revealed a significant inverse correlation between *RBM47* expression and Oxaliplatin IC50 values (r = −0.34, *p* = 0.05; Figure 6D), indicating that elevated *RBM47* expression is associated with increased Oxaliplatin sensitivity. To determine whether there is a causal relationship between RBM47 and sensitivity to Oxaliplatin, we knocked down *RBM47* in DLD1 and HCT15 cells and measured Oxaliplatin sensitivity. *RBM47* knockdown significantly increased Oxaliplatin IC50 values in both cell lines (Figure 6E and Figure S6B). Conversely, ectopic expression of RBM47 in SW480 and SW620 cells significantly decreased Oxaliplatin IC50 values (Figure 6F and Figure S6C). Notably, ectopic RBM47 expression did not alter sensitivity to 5-Fu (Figure S6D,E), indicating a specificity for Oxaliplatin-induced cell death. Similarly to *RBM47*, *GSDMA* expression showed a significant inverse correlation with Oxaliplatin IC50 values across 36 CRC cell lines (r = −0.51, *p* = 0.0037; Figure 6G). Knockdown of *GSDMA* increased Oxaliplatin IC50 values in DLD1 and HCT15 cells (Figure 6H and Figure S6F), while ectopic GSDMA expression decreased Oxaliplatin IC50 values in SW480 and SW620 cells (Figure 6I and Figure S6G). In SW480 and SW620 cells, ectopic RBM47 expression significantly enhanced Oxaliplatin sensitivity, but this effect was abolished by *GSDMA* knockdown (Figure 6J and Figure S6H). Interestingly, knockdown of *RBM47* resulted in a decreased sensitivity to Oxaliplatin, which was restored by ectopic *GSDMA* expression (Figure 6K). Taken together, these results demonstrate that RBM47 enhances chemosensitivity to Oxaliplatin via inducing *GSDMA* expression in CRC cells.

### 2.7. Characterization of RBM47-Induced GSDMA Effects on Pyroptosis

**Figure 7:**
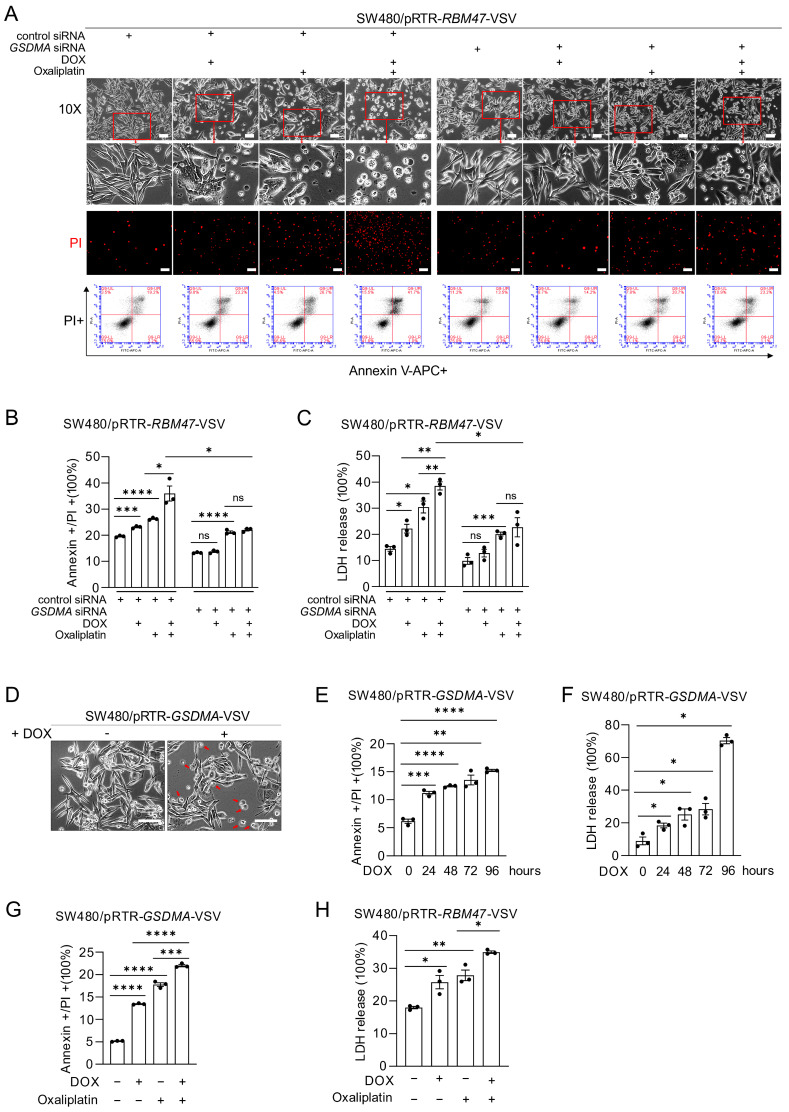
GSDMA mediates RBM47-induced pyroptosis and chemosensitization in the presence of Oxaliplatin. (**A**) Representative phase-contrast images (upper two rows), PI fluorescence images (third row), and flow cytometry analysis (bottom row) of SW480/pRTR-*RBM47*-VSV cells transfected with control or *GSDMA*-specific siRNA and treated with or without DOX and/or Oxaliplatin for 96 h. Red arrows indicate cells undergoing pyroptosis. Scale bars, 50 μm. (**B**,**C**) Quantification of Annexin V and PI double-positive cells (**B**) and LDH release (**C**) in SW480/pRTR-*RBM47*-VSV cells transfected with control or *GSDMA*-specific siRNAs and treated with indicated combinations of DOX and Oxaliplatin. (**D**) Representative phase-contrast images of SW480/pRTR-*GSDMA*-VSV cells treated with or without DOX for 96 h. Red arrows indicate cells undergoing pyroptosis. Scale bars, 50 μm. (**E**,**F**) Quantification of Annexin V and PI double-positive cells (**E**) and LDH release (**F**) in SW480/pRTR-*GSDMA*-VSV cells treated with DOX for indicated time points. (**G**) Quantification of Annexin V and PI double-positive cells in SW480/pRTR-*GSDMA*-VSV cells treated with or without DOX and/or Oxaliplatin for 96 h. (**H**) Quantification of LDH release in SW480/pRTR-*RBM47*-VSV cells treated with or without DOX and/or Oxaliplatin for 48 h. Mean values ± SD (n = 3) are provided. ns, not significant; * *p* < 0.05; ** *p* < 0.01; *** *p* < 0.001; **** *p* < 0.0001.

In cells transfected with control siRNA, ectopic *RBM47* expression induced morphological changes characteristic of pyroptosis, such as cell swelling and membrane blebbing, PI uptake, and increased Annexin V/PI double-positive cells in the presence of Oxaliplatin (Figure 7A,B). However, *GSDMA* knockdown prevented RBM47-induced pyroptosis and significantly reduced LDH release in the presence of Oxaliplatin (Figure 7A–C). Therefore, the induction of *GSDMA* by RBM47 mediates, at least in part, the sensitization towards Oxaliplatin. Ectopic expression of GSDMA alone was sufficient to induce the morphological changes typical for pyroptosis (Figure 7D) and significantly increased Annexin V/PI double-positive cells and LDH release in a time-dependent manner (Figure 7E,F and Figure S7A). These results demonstrate that elevated GSDMA expression is sufficient for inducing pyroptosis in CRC cells. Oxaliplatin treatment further enhanced pyroptosis in cells with ectopic *GSDMA* expression, as evidenced by Annexin V/PI staining and LDH release (Figure 7G,H and Figure S7B). Taken together, these results demonstrate that *GSDMA* mediates RBM47-induced pyroptosis and chemo-sensitization towards Oxaliplatin in CRC cells.

## 3. Discussion

**Figure 8:**
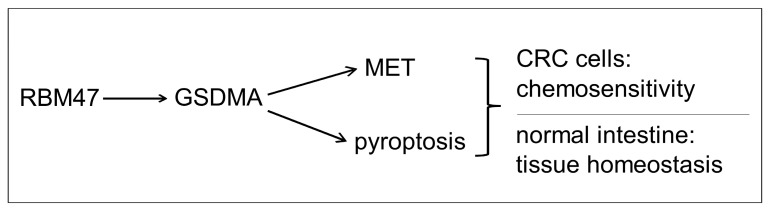
Schematic summary. Model of the results obtained in this study.

In this study, we found that GSDMA represents the most significantly induced factor downstream of RBM47 in CRC cells. *GSDMA* was the most strongly up-regulated transcript upon ectopic RBM47 expression and conversely down-regulated when RBM47 was silenced. Our findings uncover a novel RBM47/GSDMA signaling axis that links epithelial differentiation and pyroptosis with chemotherapy sensitivity (summarized in Figure 8). This work addresses a significant gap in understanding how RBM47 mediates tumor suppression, as prior studies had established RBM47 as an antagonist of EMT and metastasis but had not pinpointed its critical effectors [8].

Since RBM47 represents an RNA-binding protein, the mechanism leading to elevated abundance of the *GSDMA* mRNA is most likely the binding of RBM47 to the *GSDMA* mRNA. Further studies are needed to explore alternative mechanisms, such as transcriptional activation of *GSDMA* by RBM47. Interestingly, it has been reported that RBM47 may function as a transcription factor [36,37,38].

In colorectal cancer cells, the activation of GSDMA may promote pyroptosis, which is a lytic form of inflammatory, programmed cell death executed by the gasdermin family of pore-forming proteins [39,40]. Unlike apoptosis, which maintains membrane integrity and generates immunological tolerance, pyroptosis is characterized by rapid plasma membrane rupture and uncontrolled release of intracellular contents [41,42]. GSDMA, which is preferentially expressed in gastrointestinal epithelium [27], can be activated through proteolytic cleavage by bacterial proteases or through phosphorylation-dependent mechanisms involving ULK1 kinase during metabolic stress [43]. Upon activation, the N-terminal pore-forming domain of GSDMA oligomerizes in the plasma membrane and mitochondrial membranes, forming pores that enable release of the pro-inflammatory cytokines IL-1β and IL-18, as well as damage-associated molecular patterns (DAMPs) including HMGB1 and ATP [44,45]. In colorectal cancer, *GSDME* (*DFNA5*) is silenced through promoter hypermethylation in 34–65% of all cases [46,47]. Functional studies demonstrate that restoring GSDME expression inhibits cell proliferation and colony formation, indicating its tumor-suppressive role [46]. The silencing of gasdermins enables CRC cells to undergo immunologically silent apoptosis rather than immunogenic pyroptosis in response to chemotherapy or immune attack [33,48]. Therefore, therapeutic reactivation of *RBM47* and/or *GSDMA* in CRC may be advantageous in the context of therapies based on directing the immune system against tumor cells, such as immune-check-point inhibition. It has been shown that Oxaliplatin exhibits immune-modulatory effects and synergizes with immune-checkpoint inhibition in CRC and other cancers [49,50]. We showed that RBM47 and GSDMA increase the sensitivity towards Oxaliplatin. Therefore, *RBM47* and/or *GSDMA* might contribute to the immune-modulatory properties of Oxaliplatin by inducing immunogenic pyroptosis. Interestingly, in esophageal cancer, the suppression of GSDMA also reduced the sensitivity to Cisplatin [28], which also conveys immune-modulating effects [51].

The observed increased sensitivity against Oxaliplatin observed here may have multiple reasons. It is well known, that cancer cells in a mesenchymal-like state exhibit enhanced resistance towards chemotherapeutic drugs and cellular stressors in general. Since the promotion of MET by RBM47 and/or GSDMA promotes the acquisition of an epithelial-like state of CRC cells, their relative sensitivity to Oxaliplatin and similar drugs may be increased. Alternatively, the enhancement of the pyroptotic processes by elevated GSDMA levels may lead to an increased sensitivity towards drugs, such as Oxaliplatin. Future studies should uncover how the mechanisms underlying the increased sensitivity of RBM47-expressing cells detected here are interconnected. A full understanding of the involved mechanisms may also allow us to understand how the commonly observed down-regulation of *RBM47* in CRC and other entities contributes to chemoresistance. The *RBM47* promoter is frequently methylated in metastatic CRC, which is associated with low RBM47 protein expression [12]. As shown here, low *RBM47* expression is associated with Oxaliplatin and/or chemoresistance. Therefore, *RBM47* promoter methylation could represent a potential biomarker for resistance, whereas elevated RBM47 expression in CRCs may predict sensitivity towards Oxaliplatin and/or chemotherapy.

## 4. Materials and Methods

### 4.1. Cell Culture, Treatment, and Transfections

SW480, SW620, DLD1, and HCT15 colorectal cancer cell lines (authenticated by STR analysis (Eurofins)) were propagated in McCoy’s 5A medium (Invitrogen) supplemented with 10% fetal bovine serum (FBS; Invitrogen), penicillin (100 U/mL), and streptomycin (0.1 mg/mL). Cells were cultured at 37 °C in a humidified atmosphere containing 5% CO_2_. Oxaliplatin (Sigma-Aldrich, Saint Louis, MO, USA, O9512) stocks (5 mM) were prepared in sterile water and stored at −80 °C. 5-Fluorouracil (Sigma-Aldrich, Saint Louis, MO, USA, 343922) was dissolved in DMSO (5 mg/mL). Nigericin (InvivoGEN, San Diego, CA, USA, CAS 28643-80-3) was prepared in absolute ethanol (5 mM) and stored at −20 °C. Working solutions were freshly diluted in complete medium. siRNAs and negative controls were transfected at a final concentration of 10 nM using Lipofectamine RNAiMAX Transfection Reagent (Invitrogen, Carlsbad, CA, USA) according to the manufacturer’s instructions. Gene-specific siRNAs targeting GSDMA (FlexiTube siRNA pool, Qiagen, Hilden, Germany, Cat. No. 1027416) and non-targeting controls were obtained from Qiagen. Additionally, siRNAs against RBM47 (Silencer Select, ID# s29090) and corresponding negative controls (ID# 4611) were purchased from Invitrogen, Carlsbad, CA, USA.

### 4.2. RNA Extraction and Quantitative Real-Time PCR (qPCR) Analysis

Total RNA was purified using the High Pure RNA Isolation Kit (Roche, Grenzach-Wyhlen, Germany). One microgram of RNA was reverse-transcribed with the Verso cDNA Synthesis Kit (Thermo Fisher Scientific, Waltham, MA, USA). Quantitative PCR was carried out on a LightCycler 480 (Roche) using Fast SYBR Green Master Mix (Applied Biosystems, Carlsbad, CA, USA). *GAPDH* or *ACTIN* served as the internal reference gene, and relative expression was computed using the 2^−ΔΔCt^ method [52]. Expression values were normalized to the corresponding control group and are presented as fold changes. All reactions were performed in technical triplicate to ensure reproducibility. Primer sequences used for amplification are listed in the Supplemental Table S2.

### 4.3. Establishment of DOX-Inducible pRTR-RBM47-VSV and pRTR-GSDMA-VSV Pools

Inducible expression pools were generated using the pRTR doxycycline-responsive system [53]. The coding sequence (CDS) of human *RBM47* was PCR-amplified from the pEGFP-N1-Flag-human *RBM47* construct (Addgene, Watertown, MA, USA, Plasmid #264557), and the CDS of human *GSDMA* was obtained from the *GSDMA*_OHu07468D_pcDNA3.1+/C-(K)-DYK plasmid (GenScript, Piscataway, NJ, USA, Cat. No. OHu07468D). The amplified CDS fragments were first cloned into a modified shuttle vector, pUC19-SfiI-CVSV, via SfiI restriction sites. After enzymatic release, the inserts were subcloned into the pRTR backbone. Correct orientation and open reading frame (ORF) integrity were confirmed by Sanger sequencing. For stable transgene integration, cells were transfected with the pRTR-*RBM47*-VSV or pRTR-*GSDMA*-VSV constructs using Lipofectamine^®^ LTX & PLUS™ Reagent (Invitrogen), following the manufacturer’s instructions. Transfected cells were selected with puromycin (8 μg/mL), which was replenished with fresh culture medium every 48 h for a 14-day selection period. To induce transgene expression, DOX was freshly dissolved in sterile water at a stock concentration of 100 μg/mL and applied to cultures at a final concentration of 100 ng/mL. The uniformity and inducibility of the transgene-expressing cell pools were assessed by treating cells with DOX for 48 h, followed by flow cytometric analysis of GFP expression driven by the pRTR vector.

### 4.4. Western Blot Analysis

Cells were lysed on ice in RIPA buffer (50 mM Tris-HCl, pH 8.0; 1% NP-40; 250 mM NaCl; 0.5% sodium deoxycholate; 0.1% SDS) supplemented with protease and phosphatase inhibitors (Roche). Lysates were sonicated (5 s per sample, 70% amplitude) and cleared by centrifugation (13,000× *g*, 20 min, 4 °C). Protein concentration was determined by BCA assay (Thermo Fisher Scientific). Equal protein (40 μg) was resolved on 10–12% SDS–PAGE and transferred to PVDF membranes (Immobilon-P, Millipore, Burlington, MA, USA) using a Trans-Blot Turbo system (Bio-Rad, Hercules, CA, USA). Membranes were blocked in 5% milk/TBST for 1 h, incubated with primary antibodies (Supplementary Table S1) overnight at 4 °C, and then with HRP-conjugated secondary antibodies for 1 h at room temperature. Protein bands were visualized using ECL substrate (Cytiva, Marlborough, MA, USA) and imaged with an Odyssey^®^ FC Imaging System (LI-COR Biosciences, Courne, MA, USA). Band intensities were quantified using Image Studio™ software v5.2.

### 4.5. Confocal Imaging and Morphology Assessment

Cells were plated in 6-well plates and analyzed at 40–60% confluence following the indicated treatments. Morphological phenotypes were documented on a Zeiss LSM 700 confocal microscope (Zeiss, Oberkochen, Germany); image acquisition and processing were performed using ZEN 3.11 software.

### 4.6. Structure-Based Interaction Prediction

RBM47-GSDMA 3′ untranslated region (3′UTR) binding interactions were predicted using the AlphaFold3 server (DeepMind, https://alphafoldserver.com (accessed on 1 March 2024)). The server-reported confidence metrics were used to rank models, and recurrent protein–RNA contact regions among top-ranked predictions were extracted as candidate binding segments for downstream experimental validation. No quantitative binding affinity was inferred from these in silico models.

### 4.7. RNA-Binding Protein Immunoprecipitation (RIP)

In compliance with the manufacturer’s instructions, the Magna RIP Kit (Sigma-Aldrich, Saint Louis, MO, USA, Catalog No. 17-700) was utilized for performing RIP analysis. Briefly, SW480/pRTR-*RBM47*-VSV cells were lysed in RIP lysis buffer supplemented with protease and RNase inhibitors. Cell lysates were incubated overnight at 4 °C with magnetic beads conjugated to anti-VSV antibody and anti-RBM47 antibody to immunoprecipitate RBM47-associated RNA complexes. A parallel immunoprecipitation using normal IgG served as a negative control. The beads were then washed three times, and Chloroform-isopropanol reagent was used to extract the RNA in the immunoprecipitates and 10% inputs. The RT-qPCR was conducted to quantify *GSDMA* mRNA.

### 4.8. Immunofluorescence (If) Analysis

For detection by indirect immunofluorescence, cells were cultured on sterile glass coverslips in 12-well plates until reaching 70–80% confluence. Following experimental treatments, cells were fixed with 4% paraformaldehyde (PFA) for 15 min at room temperature, then permeabilized using 0.1% Triton X-100 (The Dow Chemical Company, Zaozhuang, China) for 10 min. Non-specific binding was blocked with 1% BSA in PBS for 1 h. Cells were incubated with primary antibodies (listed in Supplementary Table S1) overnight at 4 °C, followed by Alexa Fluor–conjugated secondary antibodies for 1 h at room temperature. Nuclei were counterstained with DAPI (Roche) for 5 min. Coverslips were mounted onto glass slides using ProLong™ Gold Antifade Reagent (Invitrogen). Fluorescence images were acquired using either a confocal laser scanning microscope (LSM 700, Zeiss) operated with ZEN 2009 software, or a PhenoImager^®^ HT whole-slide multispectral imaging system (Akoya Biosciences, Marlborough, MA, USA) operated with HT 2.0 software. Within each experiment, identical acquisition settings were maintained across conditions for each platform. Fluorescence quantification was normalized per cell using DAPI-based nuclear segmentation. Raw image files were exported and converted to TIFF format for subsequent analysis.

### 4.9. Propidium Iodide (PI) Staining

Cells cultured in 24-well imaging plates were subjected to the indicated treatments and then incubated with PI (1 µg/mL; Sigma-Aldrich, Saint Louis, MO, USA) for 20–30 min at 37 °C in the dark. Samples were imaged live on a Zeiss LSM 700 confocal microscope.

### 4.10. Cell Viability Assay

Cell viability was determined using CCK-8 reagent (CK-8; Dojindo, Munich, Germany, Cat. No. CK04). Cells (3000/well) were seeded in 96-well plates and treated as indicated. CCK-8 solution (10 μL) was added for 2 h at 37 °C, and absorbance was measured using a microplate reader at 450 nm. Viability was calculated relative to untreated controls.

### 4.11. RNA Decay Assay

Cells were seeded in a 6-well plate at a density of 1 × 10^5^ cells per well and cultured to approximately 80% confluence. Transcription was inhibited by adding actinomycin D (5 μg/mL; Sigma-Aldrich, Saint Louis, MO, USA) directly to the culture medium. Total RNA was extracted at the indicated time points (0, 2, 4, 6, and 8 h), followed by reverse transcription and qPCR as described above. Relative transcript abundance at each time point was normalized to *GAPDH* expression. mRNA half-life was determined by fitting the decay curves to a first-order exponential regression model using GraphPad Prism 10.04 software.

### 4.12. Real-Time Cell Proliferation/Impedance Assay (xCELLigence)

Cell growth dynamics were monitored using impedance-based real-time analysis (xCELLigence RTCA DP, Acea Biosciences, San Diego, CA, USA). Cells (3000/well, in 100 μL medium) were seeded in E-Plate 16 wells containing gold microelectrodes and allowed to adhere for 24 h before treatment. Electrical impedance was recorded hourly for 140 h and automatically converted to Cell Index values using RTCA software v1.0. End-point cell counts in parallel-seeded 96-well plates were performed to validate impedance-based measurements.

### 4.13. Transwell Migration and Invasion Assays

Cell migration and invasion were assessed using 24-well Transwell chambers (8 μm pore size, Corning, Corning, NY, USA). For migration assays, serum-starved cells (1 × 10^5^) in serum-free medium were seeded in upper chambers with complete medium containing 10% FBS as chemoattractant in lower chambers. For invasion assays, membranes were pre-coated with diluted Matrigel (1:6, BD Biosciences, San Jose, CA, USA) for 2 h at 37 °C before cell seeding. Cells were incubated for 24 h (migration) or 48 h (invasion). Non-migrated/invaded cells on upper surfaces were removed using cotton swabs, and chambers were fixed with methanol for 30 min. Cells were stained with 0.5% crystal violet for 30 min and rinsed in PBS. Images from three random microscopic fields per insert were captured, and migrated or invaded cells were quantified using ImageJ v1.54p software.

### 4.14. Annexin V/PI Flow Cytometry

Cell death profiles were assessed using Annexin V APC/PI dual staining (BD Biosciences). Following treatment, cells were harvested by trypsinization, washed twice with cold PBS, and resuspended in 1× binding buffer at 1 × 10^6^ cells/mL. Cell suspensions (100 μL, containing 1 × 10^5^ cells) were incubated with Annexin V APC and PI for 15 min at room temperature in the dark. After adding 400 μL binding buffer, samples were analyzed immediately on a Accuri™ C6 Plus flow cytometer (BD, Franklin Lakes, NJ, USA). Annexin V^+^/PI^−^ (early apoptotic), Annexin V^+^/PI^+^ (late-stage membrane-compromised cell death), and Annexin V^−^/PI^+^ (primary necrosis-like) populations were quantified using BD Accuri C6 Plus software, with ≥10,000 events acquired per sample.

### 4.15. Cell Cycle Analysis

Cell cycle distribution was determined by PI staining. Cells (2 × 10^5^/well) were seeded in 6-well plates and treated as indicated. Both floating and adherent cells were harvested, washed with PBS, and fixed with ice-cold 70% ethanol at −20 °C overnight. Fixed cells were washed and resuspended in PI staining solution containing RNase A, then analyzed using BD Accuri™ C6 Plus flow cytometer.

### 4.16. Lactate Dehydrogenase (LDH) Release Assay

Cell membrane integrity was assessed by measuring LDH release using the Cytotoxicity LDH Assay Kit (MedChemExpress, Monmouth Junction, NJ, USA, Cat. No. HY-K1090) according to the manufacturer’s protocol. After treatment, culture supernatants were collected and incubated with the LDH reaction mixture. Absorbance was measured at 490 nm using a microplate reader.

### 4.17. RNA Isolation

Total RNA was isolated from DLD1 cells 72 h after transfection with control or RBM47 siRNA, and from SW480/pRTR-RBM47-VSV cells treated with vehicle or DOX for 24 h and 96 h. Each condition was performed in biological triplicate. RNA quality was assessed using a Bioanalyzer (Agilent Technologies, Santa Clara, CA, USA).

### 4.18. mRNA Expression Profiling by RNA-seq and Bioinformatics Analyses

RNA profiling was performed in triplicate. Random primed complementary DNA libraries were constructed and sequenced using the HiSeq2500 (Illumina, San Diego, CA, USA) platform by Eurofins/GATC. Each sample was covered by at least 30 million single reads of 50 bp length. The raw reads (FASTQ files) were cleaned by trimming adaptor sequences and low-quality sequences with average quality scores < 20. Trimmed reads were mapped to the hg38 human reference genome and processed using the RNA-seq module of the CLC Genomics Workbench v20.0 software (Qiagen Bioinformatics, Hilden, Germany) with default settings. RNA-seq data were filtered to exclude weakly expressed transcripts with less than ten mapped reads in all samples from the analysis and subjected to normalization using the R-package RUVSeq (remove unwanted variation from RNA-seq data) package. Differential gene expression analysis was performed using the limma method. Gene set enrichment analyses (GSEA) were performed using GSEA software (Broad Institute, Boston, MA, USA) and data from the molecular signature database (MSigDB). The significance of enrichments is presented by normalized enrichment scores with false discovery rate–adjusted q values. Heatmaps were generated with Morpheus (Broad Institute, Boston, MA, USA).

### 4.19. Statistical Analysis

Data representation follows the mean ± standard deviation format, derived from at least three independent biological replicates. Normality assumptions were tested via Shapiro–Wilk analysis. For normally distributed data, two-group comparisons employed unpaired two-tailed Student’s *t*-tests, while multi-group comparisons utilized one-way ANOVA followed by Tukey’s post hoc test for multiple comparison corrections. Pearson correlation analysis was used to assess gene expression associations. *p*-values < 0.05 were considered statistically significant (* *p* < 0.05; ** *p* < 0.01; *** *p* < 0.001; **** *p* < 0.0001). Statistical analyses were performed using GraphPad Prism 10.04 (GraphPad Software, Boston, MA, USA).

## 5. Conclusions

Here, we demonstrated that RBM47 binds to and stabilizes the *GSDMA* mRNA. GSDMA mediated the RBM47-induced mesenchymal-to-epithelial transition as well as suppression of migration and invasion by RBM47. Furthermore, RBM47 triggered pyroptosis via GSDMA. Activation of the RBM47–GSDMA axis enhanced sensitivity to Oxaliplatin through concomitant induction of epithelial differentiation and pyroptotic cell death. Therefore, GSDMA represents a mediator of the tumor suppressive effects of RBM47 in cancer cells derived from intestinal epithelia. Overall, this study shows that the RBM47–GSDMA regulatory connection controls epithelial plasticity and pyroptosis. As both processes affect sensitivity towards Oxaliplatin, detection of RBM47 and/or GSDMA expression may harbor potential as predictive biomarkers for Oxaliplatin-based chemotherapy in CRC.

## Data Availability

All data, analytic methods, and study materials will be made available to other researchers upon reasonable request.

## Supplementary Materials

The following supporting information can be downloaded at: https://www.mdpi.com/article/10.3390/cancers18030504/s1, Figure S1. RBM47 induces GSDMA expression by stabilizing GSDMA mRNA in additional CRC cell lines; Figure S2. RBM47 induces MET and suppresses malignant phenotypes in SW620 cells; Figure S3. RBM47 induces MET through GSDMA in additional CRC cell lines; Figure S4. RBM47 suppresses cell proliferation in additional CRC cell lines; Figure S5. RBM47 induces pyroptosis cell death through GSDMA in CRC cells; Figure S6. RBM47 enhances chemosensitivity to Oxaliplatin in additional CRC cell lines; Figure S7. Flow cytometry analysis of GSDMA-induced pyroptosis-like cell death and chemosensitization; Table S1: Antibodies; Table S2: Oligonucleotides used for qPCR; Uncropped Western blot membranes.
