# Supplementary material for: RBM47-Induced Gasdermin A/GSDMA Mediates Mesenchymal–Epithelial Transition and Pyroptosis of Colorectal Cancer Cells
Source: Cancers (Basel). 2026 Feb 3;18(3):504. doi: 10.3390/cancers18030504 (PMC12897226; doi:10.3390/cancers18030504)

Supplementary information for:

RBM47-induced GDMSA Mediates Mesenchymal-Epithelial Transition and Pyroptosis of Colorectal Cancer Cells

Yuyun Du[1], Matjaz Rokavec[1], and Heiko Hermeking[1,2,3]

[1] Experimental and Molecular Pathology, Institute of Pathology, Faculty of Medicine, Ludwig-Maximilians-Universität München, Thalkirchner Strasse 36, 80337 Munich, Germany

[2] German Cancer Consortium (DKTK), Partner site Munich, 80336 Munich, Germany

[3] German Cancer Research Center (DKFZ), 69120 Heidelberg, Germany

Inventory of supplementary information

- **Fig S1.** Related to Fig. 2
- **Fig S2.** Related to Fig. 3
- **Fig S3.** Related to Fig. 4
- **Fig S4.** Related to Fig. 5
- **Fig S5.** Related to Fig. 5
- **Fig S6.** Related to Fig. 6
- **Fig S7.** Related to Fig. 7
- **Table S1.** Antibodies
- **Table S2.** Oligonucleotides used for qPCR
- **Uncropped Western blot membranes**

Fig S1. Related to Fig. 2

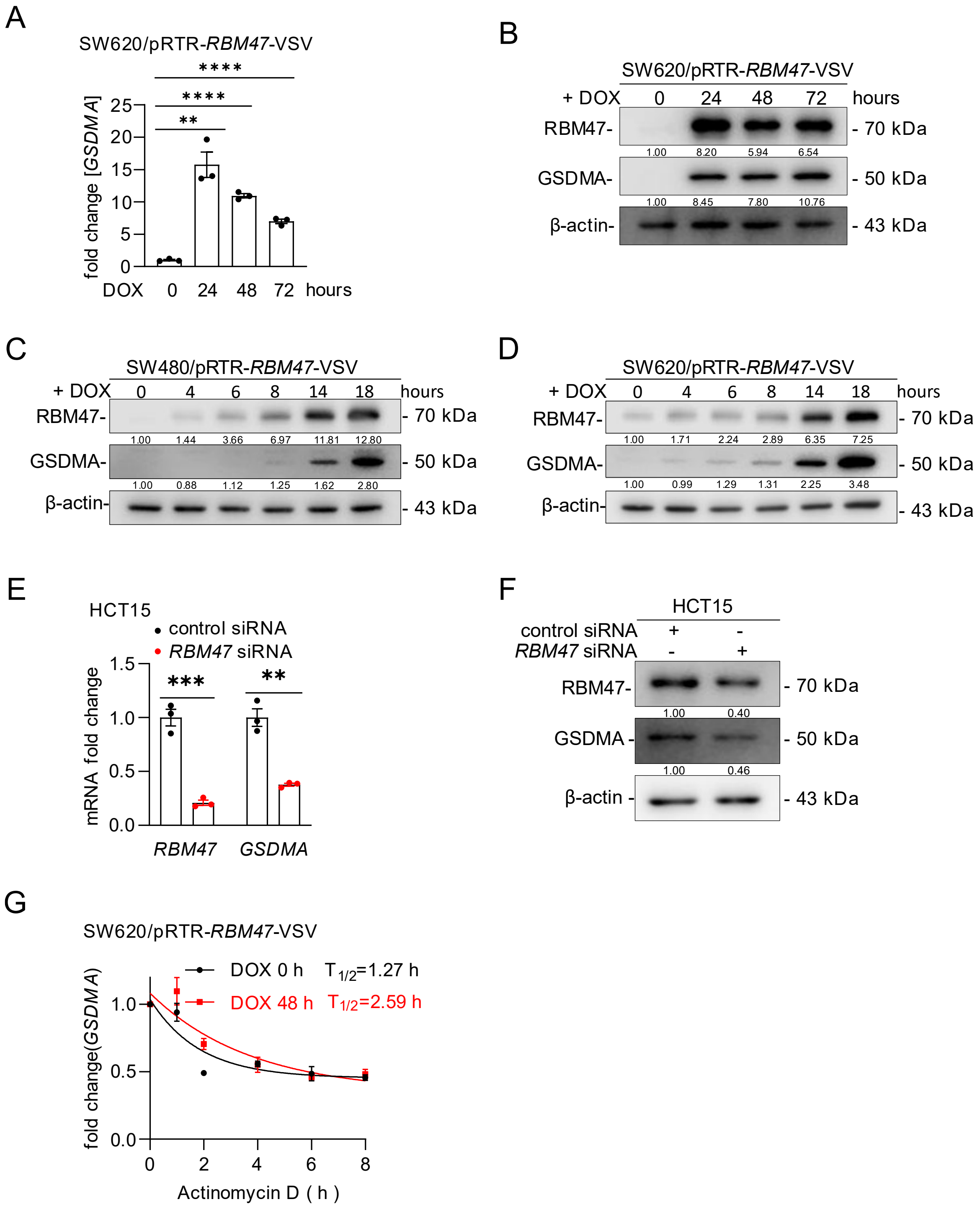

Fig S1. RBM47 induces GSDMA expression by stabilizing GSDMA mRNA in additional CRC cell lines.

(A-B) qPCR **(A)** and Western blot **(B)** analyses of *RBM47* and *GSDMA* expression in SW620/pRTR-*RBM47*-VSV cells treated with DOX for indicated time points. Numbers below Western blot bands indicate relative protein levels normalized to β-actin. **(C-D)** Western blot analyses of RBM47 and GSDMA expression in SW480/pRTR-*RBM47*-VSV **(C)** and SW620/pRTR-*RBM47*-VSV **(D)** cells treated with DOX for indicated early time points. Numbers below bands indicate relative protein levels normalized to β-actin. **(E-F)** qPCR **(E)** and Western blot **(F)** analyses of *RBM47* and *GSDMA* expression in HCT15 cells 72 hours after transfection with control or *RBM47* siRNA. Numbers below bands indicate relative protein levels normalized to β-actin. **(G)** *GSDMA* mRNA stability assay in SW620/pRTR-*RBM47*-VSV cells treated with or without DOX for 48 hours, followed by actinomycin D treatment for indicated time points. *GSDMA* mRNA half-lives ($T_1/_2$) are indicated. Mean values ± SD (n=3) are provided. ns, not significant; *P<0.05; **P<0.01; ***P<0.001; ****P<0.0001.

Fig S2. Related to Fig. 3

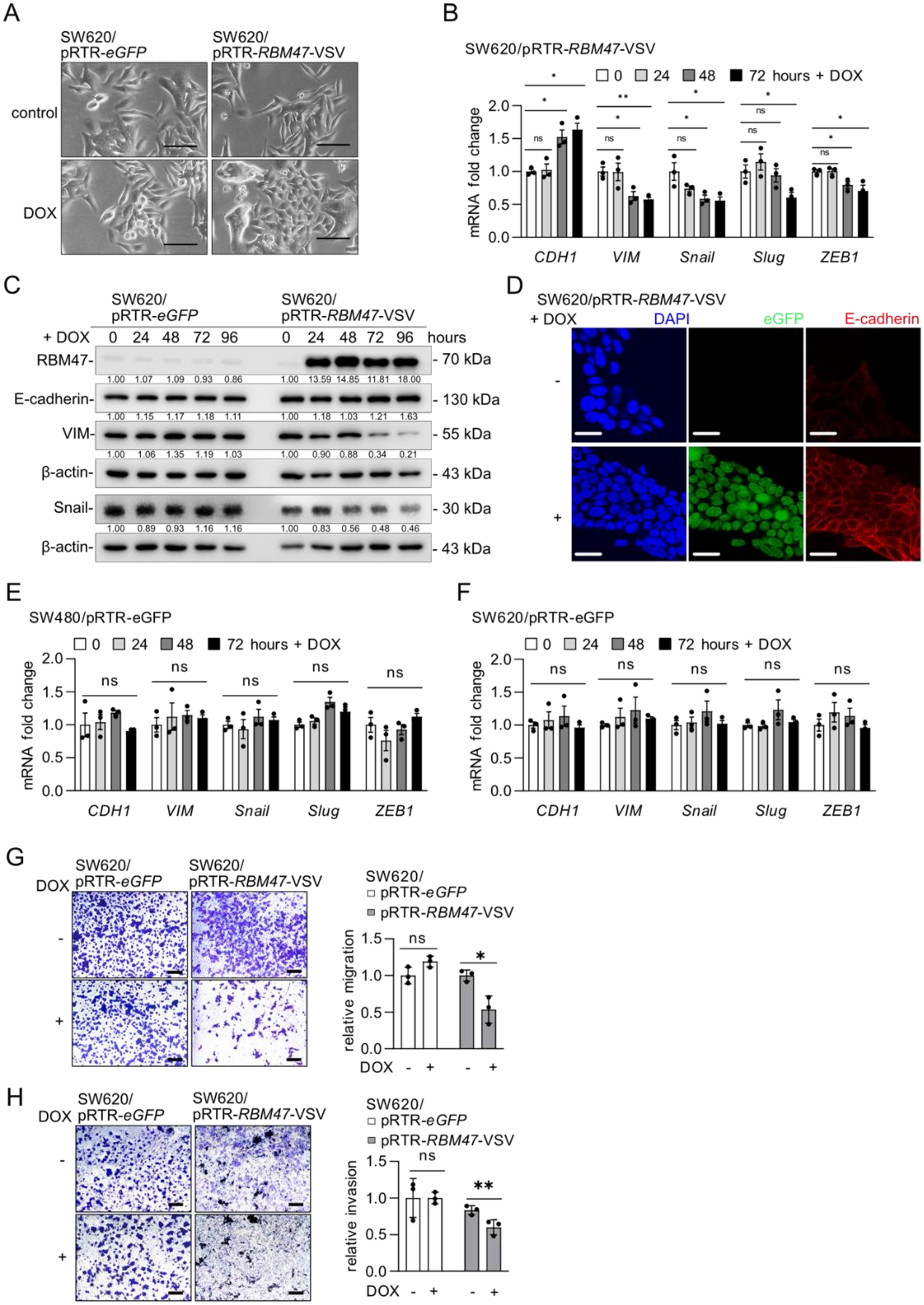

Fig S2. RBM47 induces MET and suppresses malignant phenotypes in SW620 cells. **(A)** Representative phase-contrast images of SW620/pRTR-eGFP and SW620/pRTR-*RBM47*-VSV cells treated with vehicle or DOX for 72 hours, showing morphological changes from mesenchymal to epithelial phenotype. Scale bars, 50 μm. **(B)** qPCR analysis of EMT-related genes (*CDH1*, *VIM*, *Snail*, *Slug*, and *ZEB1*) in SW620/pRTR-*RBM47*-VSV cells treated with DOX for indicated time points. **(C)** Western blot analysis of RBM47, E-cadherin, VIM, and Snail protein expression in SW620/pRTR-eGFP and SW620/pRTR-*RBM47*-VSV cells treated with DOX for indicated time points. Numbers below bands indicate relative protein levels normalized to β-actin. **(D)** Immunofluorescence staining of E-cadherin in SW620/pRTR-*RBM47*-VSV cells treated with vehicle or DOX for 72 hours. Nuclei were counterstained with DAPI. eGFP expression indicates successful vector transfection. Scale bars, 50 μm. **(E-F)** qPCR analysis of EMT-related genes (*CDH1*, *VIM*, *Snail*, *Slug*, and *ZEB1*) in SW480/pRTR-eGFP **(E)** and SW620/pRTR-eGFP **(F)** cells treated with DOX for indicated time points. **(G-H)** Representative images and quantification of Transwell migration **(G)** and invasion **(H)** assays in SW620/pRTR-eGFP and SW620/pRTR-*RBM47*-VSV cells treated with or without DOX for 48 hours. Migrated and invaded cells were stained with crystal violet. Scale bars, 50 μm. Mean values ± SD (n=3) are provided. ns, not significant; *P<0.05; **P<0.01; ***P<0.001; ****P<0.0001.

Fig S3. Related to Fig. 4

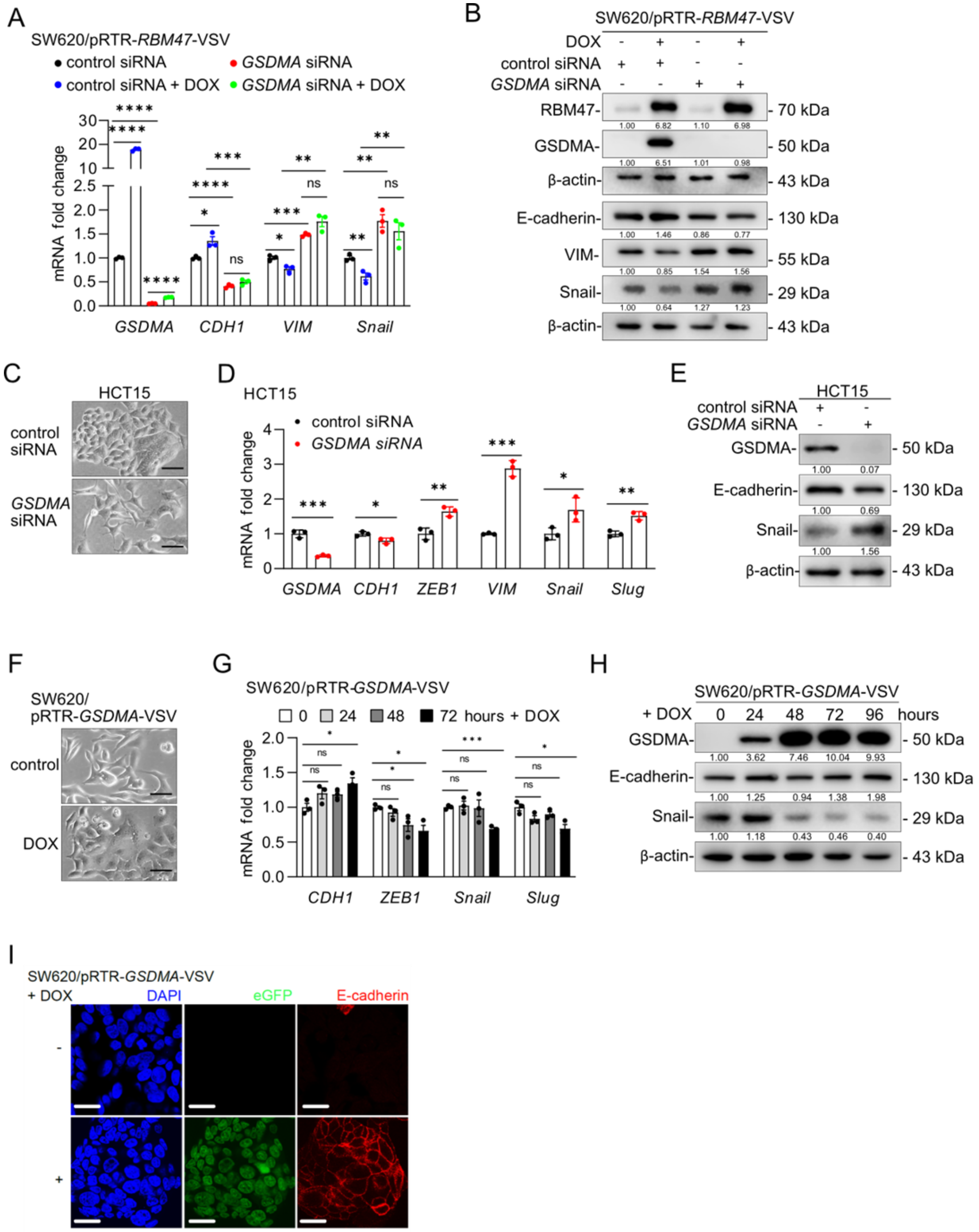

Fig S3. RBM47 induces MET through GSDMA in additional CRC cell lines.

(A-B) qPCR **(A)** and Western blot **(B)** analyses of *GSDMA* and EMT-related genes (*CDH1*, *VIM*, *Snail*) in SW620/pRTR-*RBM47*-VSV cells transfected with control or *GSDMA* siRNA and treated with or without DOX for 72 hours. Numbers below Western blot bands indicate relative protein levels normalized to β-actin. **(C)** Representative phase-contrast images of HCT15 cells 72 hours after transfection with control or *GSDMA* siRNA. Scale bars, 50 μm. **(D)** qPCR analysis of *GSDMA* and EMT-related genes (*CDH1*, *ZEB1*, *VIM*, *Snail*, *Slug*) in HCT15 cells 72 hours after transfection with control or *GSDMA* siRNA. **(E)** Western blot analysis of GSDMA, E-cadherin, and Snail protein expression in HCT15 cells 72 hours after transfection with control or *GSDMA* siRNA. Numbers below bands indicate relative protein levels normalized to β-actin. **(F)** Representative phase-contrast images of SW620/pRTR-*GSDMA*-VSV cells treated with vehicle or DOX for 72 hours. Scale bars, 50 μm. **(G-H)** qPCR **(G)** and Western blot **(H)** analyses of EMT-related genes (*CDH1*, *ZEB1*, *Snail*, *Slug*) and proteins (E-cadherin, Snail) in SW620/pRTR-*GSDMA*-VSV cells treated with DOX for indicated time points. Numbers below Western blot bands indicate relative protein levels normalized to β-actin. **(I)** Immunofluorescence staining of E-cadherin in SW620/pRTR-*GSDMA*-VSV cells treated with vehicle or DOX for 72 hours. Nuclei were counterstained with DAPI. eGFP expression indicates successful vector transfection. Scale bars, 50 μm. Mean values ± SD (n=3) are provided. ns, not significant; *P<0.05; **P<0.01; ***P<0.001; ****P<0.0001.

Fig S4. Related to Fig. 5

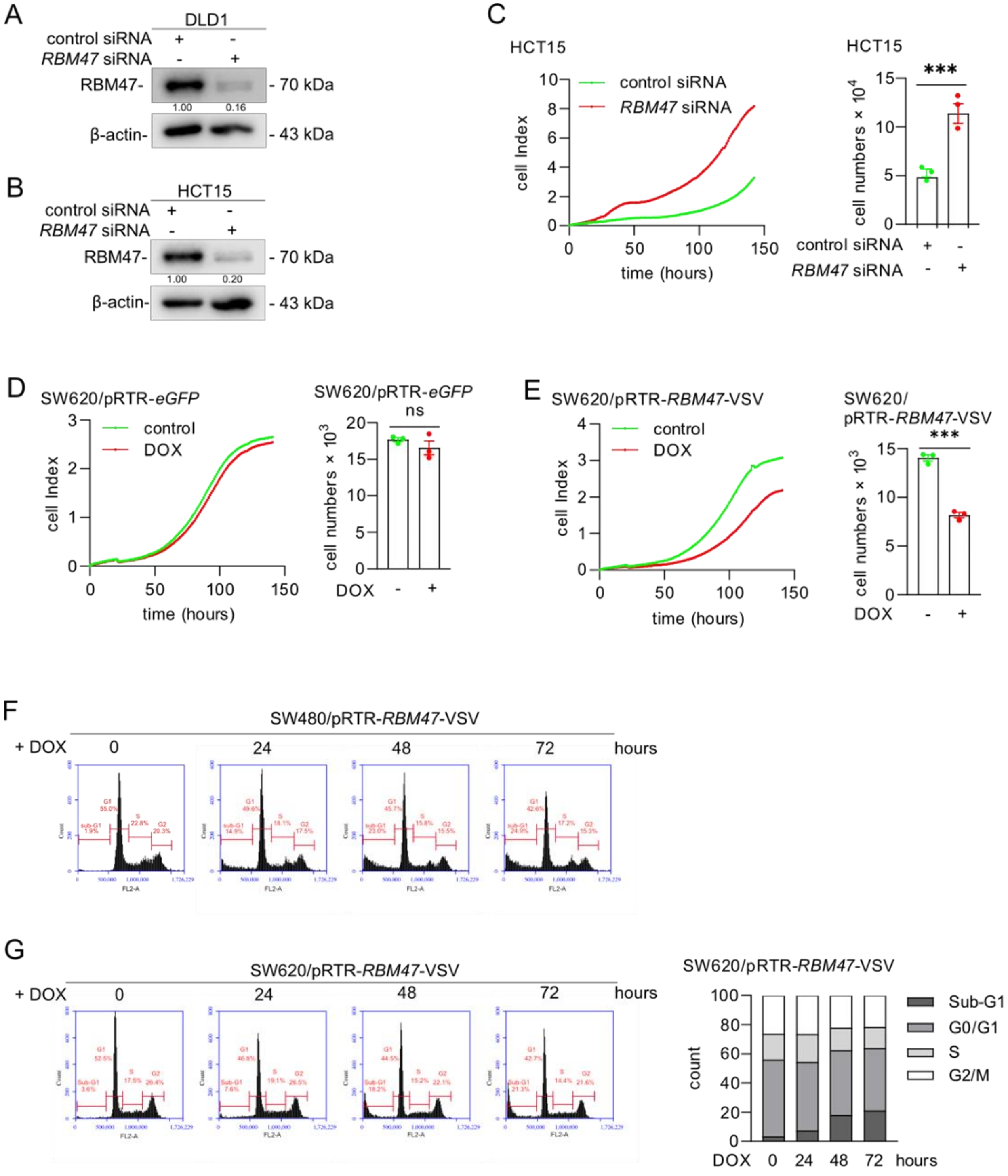

Fig S4. RBM47 suppresses cell proliferation in additional CRC cell lines.

(A-B) Western blot analysis confirming *RBM47* knockdown efficiency in DLD1 **(A)** and HCT15 **(B)** cells 72 hours after transfection with control or *RBM47* siRNA. Numbers below bands indicate relative protein levels normalized to β-actin. **(C)** Cell proliferation curves (left) and cell number quantification (right) of HCT15 cells transfected with control or *RBM47* siRNA. **(D)** Cell proliferation curves (left) and cell number quantification (right) of SW620/pRTR-eGFP cells treated with or without DOX. Cell index was measured by real-time cell analysis. **(E)** Cell proliferation curves (left) and cell number quantification (right) of SW620/pRTR-*RBM47*-VSV cells treated with or without DOX. Cell index was measured by real-time cell analysis. **(F)** Flow cytometry analysis of cell cycle distribution in SW480/pRTR-*RBM47*-VSV cells treated with DOX for indicated time points. **(G)** Flow cytometry analysis (left) and quantification (right) of cell cycle distribution in SW620/pRTR-*RBM47*-VSV cells treated with DOX for indicated time points. Cell cycle phases (Sub-G1, G0/G1, S, and G2/M) are indicated. Mean values ± SD (n=3) are provided. ns, not significant; *P<0.05; **P<0.01; ***P<0.001; ****P<0.0001.

Fig S5. Related to Fig. 5

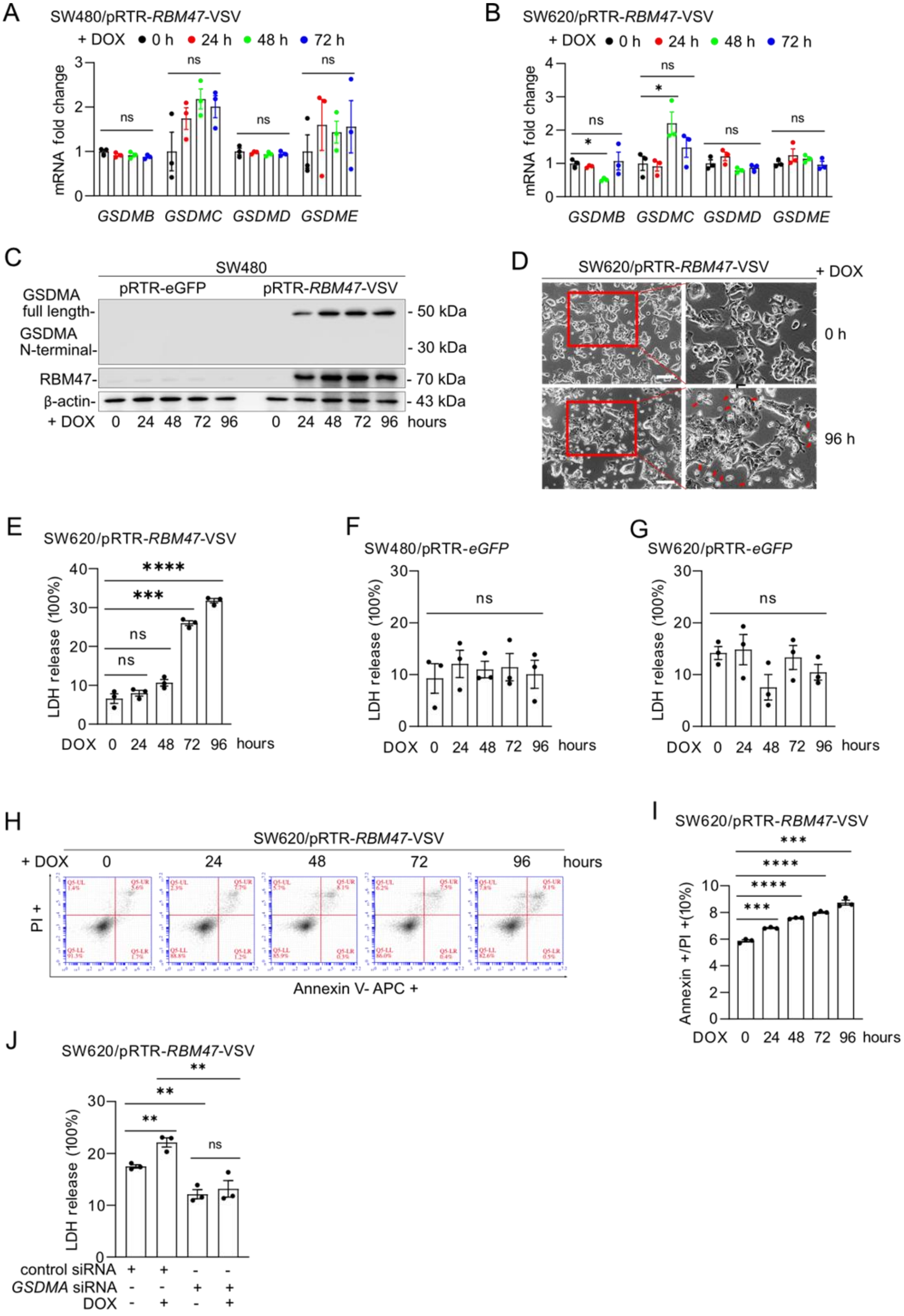

Fig S5. RBM47 induces pyroptosis cell death through GSDMA in CRC cells. (A-B) qPCR analysis of other gasdermin family members (*GSDMB*, *GSDMC*, *GSDMD*, *GSDME*) in SW480/pRTR-*RBM47*-VSV **(A)** and SW620/pRTR-*RBM47*-VSV **(B)** cells treated with DOX for indicated time points. **(C)** Western blot analysis of GSDMA full-length and N-terminal cleavage product in SW480/pRTR-eGFP and SW480/pRTR-*RBM47*-VSV cells treated with DOX for indicated time points. **(D)** Representative phase-contrast images of SW620/pRTR-*RBM47*-VSV cells treated with DOX for 0 and 96 hours. Red arrows indicate cells undergoing pyroptosis-like cell death. Scale bars, 50 μm. **(E)** Quantification of LDH release in SW620/pRTR-*RBM47*-VSV cells treated with DOX for indicated time points. **(F-G)** Quantification of LDH release in SW480/pRTR-eGFP **(F)** and SW620/pRTR-eGFP **(G)** cells treated with DOX for indicated time points. **(H-I)** Flow cytometry analysis **(H)** and quantification **(I)** of Annexin V and PI double-positive cells in SW620/pRTR-*RBM47*-VSV cells treated with DOX for indicated time points. **(J)** Quantification of LDH release in SW620/pRTR-*RBM47*-VSV cells transfected with control or *GSDMA* siRNA and treated with or without DOX for 96 hours. Mean values ± SD (n=3) are provided. ns, not significant; *P<0.05; **P<0.01; ***P<0.001; ****P<0.0001.

Fig S6. Related to Fig. 6

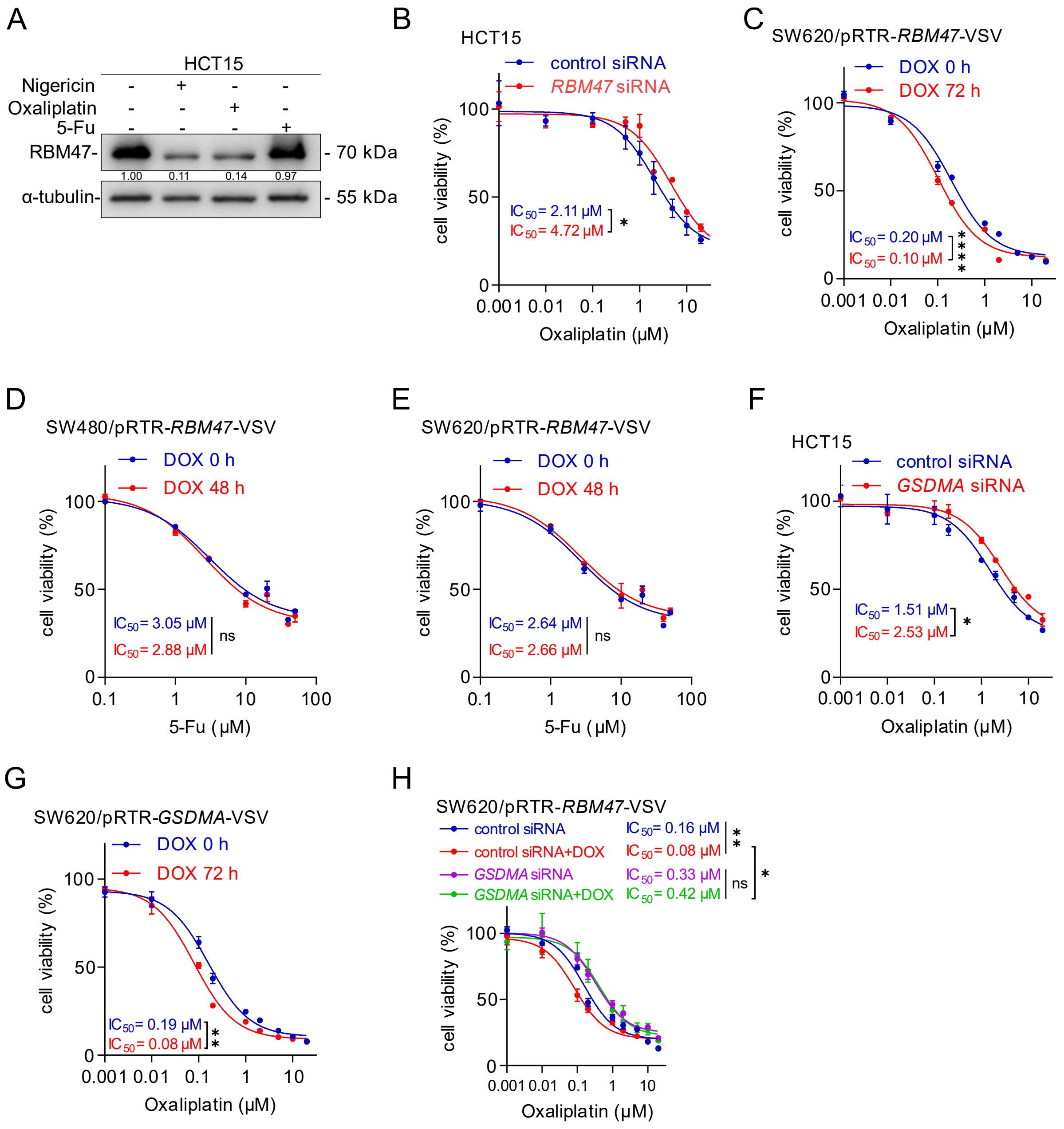

Fig S6. RBM47 enhances chemosensitivity to oxaliplatin in additional CRC cell lines. **(A)** Western blot analysis of RBM47 protein expression in HCT15 cells treated with nigericin (2 μM), oxaliplatin (5 μM), or 5-Fu (8 μM) for 48 hours. Numbers below bands indicate relative protein levels normalized to α-tubulin. **(B)** Cell viability curves of HCT15 cells transfected with control or *RBM47* siRNA and treated with increasing concentrations of oxaliplatin for 72 hours. IC50 values are indicated. **(C)** Cell viability curves of SW620/pRTR-*RBM47*-VSV cells treated with or without DOX for 72 hours, followed by oxaliplatin treatment at increasing concentrations for 72 hours. IC50 values are indicated. **(D-E)** Cell viability curves of SW480/pRTR-*RBM47*-VSV **(D)** and SW620/pRTR-*RBM47*-VSV **(E)** cells treated with or without DOX for 48 hours, followed by 5-Fu treatment at increasing concentrations for 72 hours, showing no significant change in IC50 values. **(F)** Cell viability curves of HCT15 cells transfected with control or *GSDMA* siRNA and treated with increasing concentrations of oxaliplatin for 72 hours. IC50 values are indicated. **(G)** Cell viability curves of SW620/pRTR-*GSDMA*-VSV cells treated with or without DOX for 72 hours, followed by oxaliplatin treatment at increasing concentrations for 72 hours. IC50 values are indicated. **(H)** Cell viability curves of SW620/pRTR-*RBM47*-VSV cells transfected with control or *GSDMA* siRNA, treated with or without DOX, and exposed to increasing concentrations of oxaliplatin for 72 hours. IC50 values are indicated. Mean values ± SD (n=3) are provided. ns, not significant; *P<0.05; **P<0.01; ***P<0.001; ****P<0.0001.

Fig S7. Related to Fig. 7

A

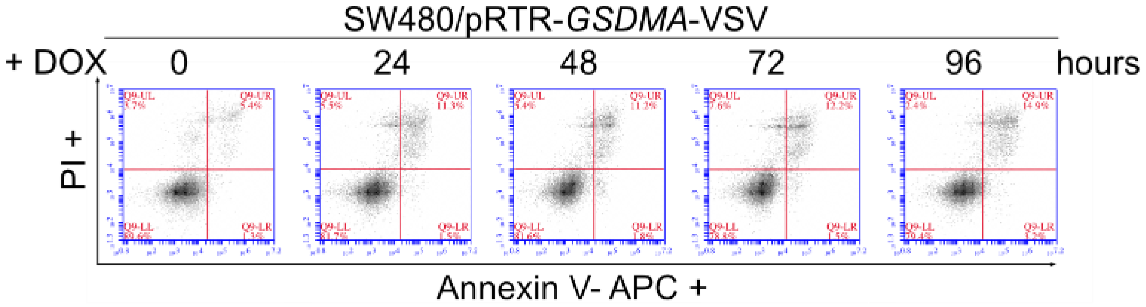

B

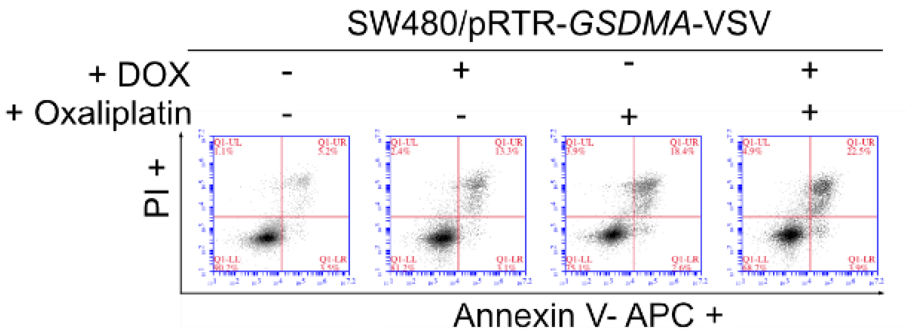

Fig S7. Flow cytometry analysis of GSDMA-induced pyroptosis-like cell death and chemosensitization. (A) Flow cytometry analysis of Annexin V and PI double-positive cells in SW480/pRTR-*GSDMA*-VSV cells treated with DOX for indicated time points (0, 24, 48, 72, and 96 hours). **(B)** Flow cytometry analysis of Annexin V and PI double-positive cells in SW480/pRTR-*GSDMA*-VSV cells treated with or without DOX and/or oxaliplatin for 96 hours.

Table S1: Antibodies

Epitope	Species	Catalog No.	Company	Use	Dilution	Sourse
Primary antibodies						
RBM47	Human	ab167164	Abcam	WB	1:1000	rabbit
				RIP	2 μg	
GSDMA	Human	ab230768	Abcam	WB	1:1000	rabbit
				IF	1:50	rabbit
Vimentin	Human	# 5741	Cell Signaling Technology	WB	1:1000	rabbit
Snail	Human	# 3879	Cell Signaling Technology	WB	1:1000	rabbit
E-cadherin	Human	# 33-4000	Invitrogen	WB	1:1000	mouse
				IF	1:1000	mouse
β-actin	Human	# A2066	Sigma-Aldrich	WB	1:1000	rabbit
α-tubulin	Human	# T-9026	Sigma-Aldrich	WB	1:1000	mouse
Anti-VSV-G	Human	# V4888	Sigma-Aldrich	RIP	2 μg	
Second antibodies						
Anti-mouse HRP	N.A.	# W4021	Promega	WB	1:10000	goat
Anti-rabbit HRP	N.A.	# A0545	Sigma-Aldrich	WB	1:10000	goat
Alexa Fluor Plus 555	N.A.	# A32727	Invitrogen	IF	1:1000	goat
Alexa Fluor Plus 647	N.A.	# A-21245	Invitrogen	IF	1:1000	goat

Table S2: Oligonucleotides used for qPCR

mRNA	forward (5'-3')	reverse (5'-3')
RBM47	CCTCATGATGGACTTTGACG	GCGGATCTCGTAGTTGTTGAG
GSDMA	AAGCTGCTGGTGAAATCCAT	GGGGGAAAACACCCTCTTTA
GSDMB	CTGAGGCACGAATTCTCTGT	ACATGGAGCGAATGGGATAC
GSDMC	TACTGTCTCTACTCCACCTG	GAGGAGTGTGGCCTTAGGAT
GSDMD	GTGTGTCAACCTGTCTATCAAGG	CATGGCATCGTAGAAGTGGAAG
GSDME	ACATGCAGGTCGAGGAGAAGT	TCAATGACACCGTAGGCAATG
GAPDH	TGTTGCCATCAATGACCCCTT	CTCCACGACGTACTCAGCG
β-actin	TGACATTAAGGAGAAGCTGTGCTAC	GAGTTGAAGGTAGTTTCGTGGATG
ZEB1	TCAAAAGGAAGTCAATGGACAA	GTGCAGGAGGGACCTCTTTA
Slug	GGGGAGAAGCCTTTTTCTTG	TCCTCATGTTTGTGCAGGAG
CDH1	CCCGGGACAACGTTTATTAC	GCTGGCTCAAGTCAAAGTCC
Vimentin	TACAGGAAGCTGCTGGAAGG	ACCAGAGGGAGTGAATCCAG
Snail	GCACATCCGAAGCCACAC	GGAGAAGGTCCGAGCACAC

Uncropped Western blot membranes.

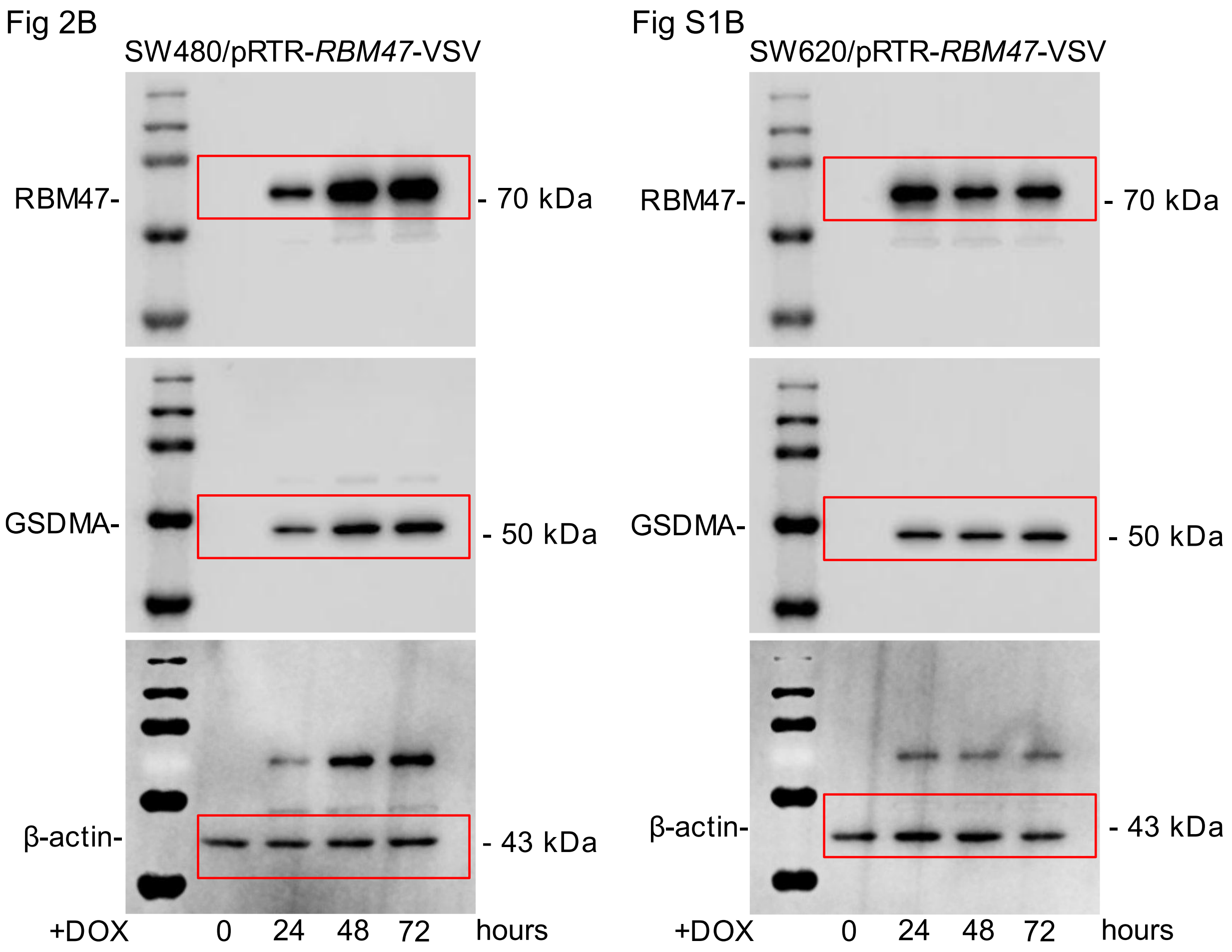

Fig 2E

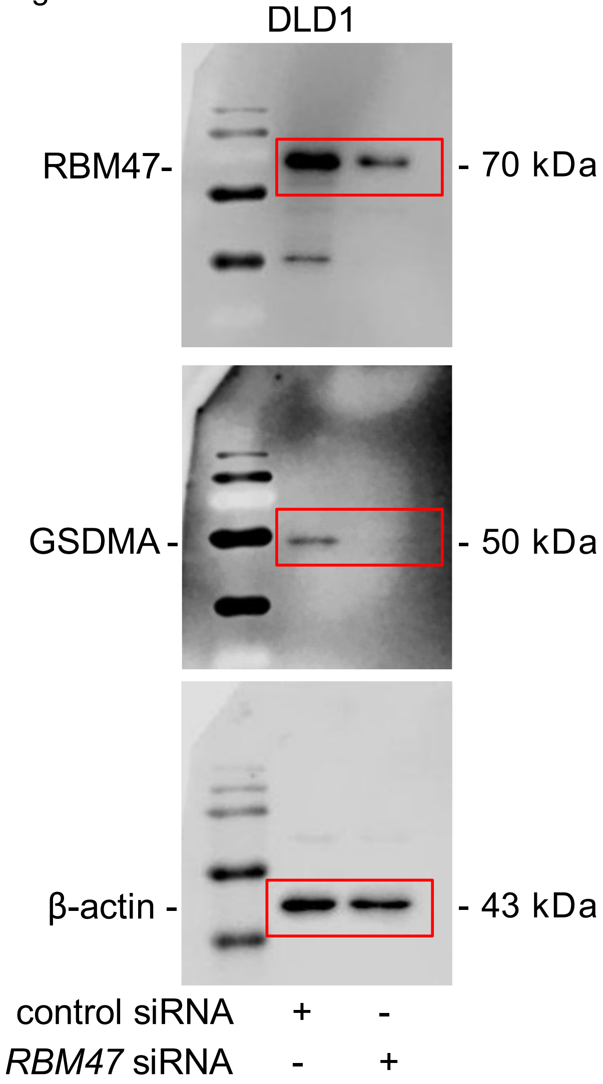

Fig S1F

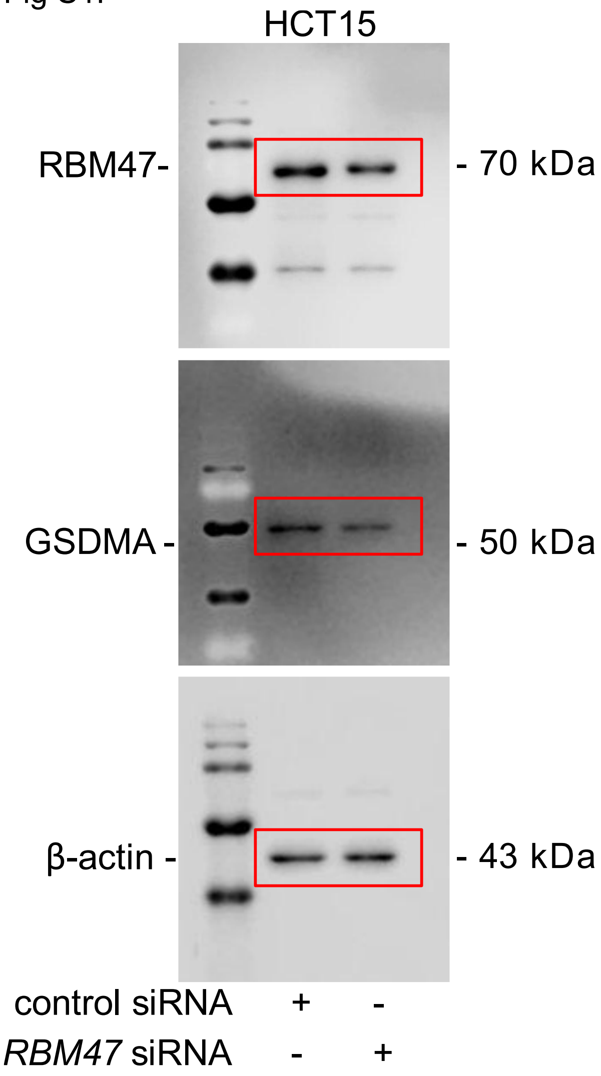

Fig S1C

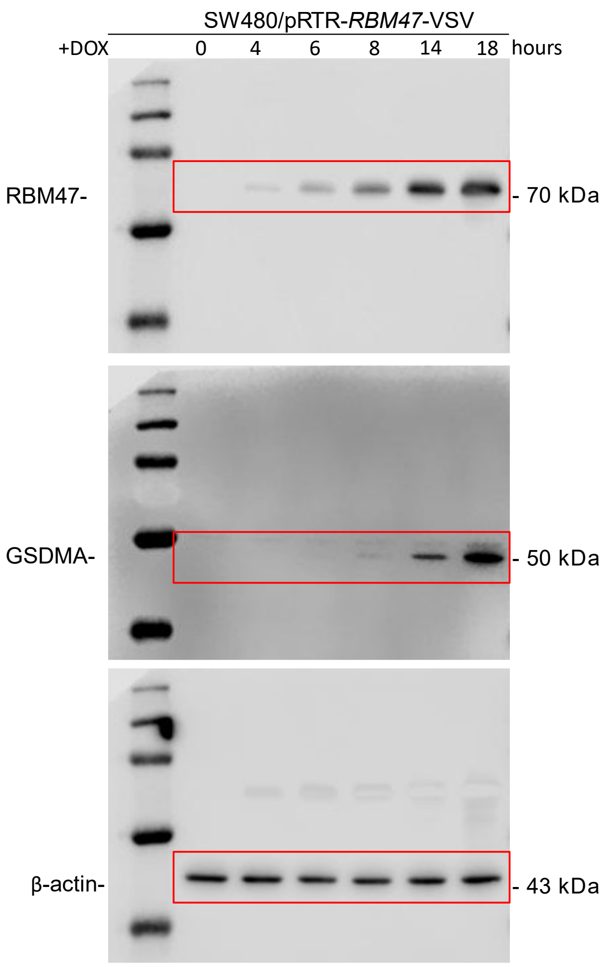

Fig S1D

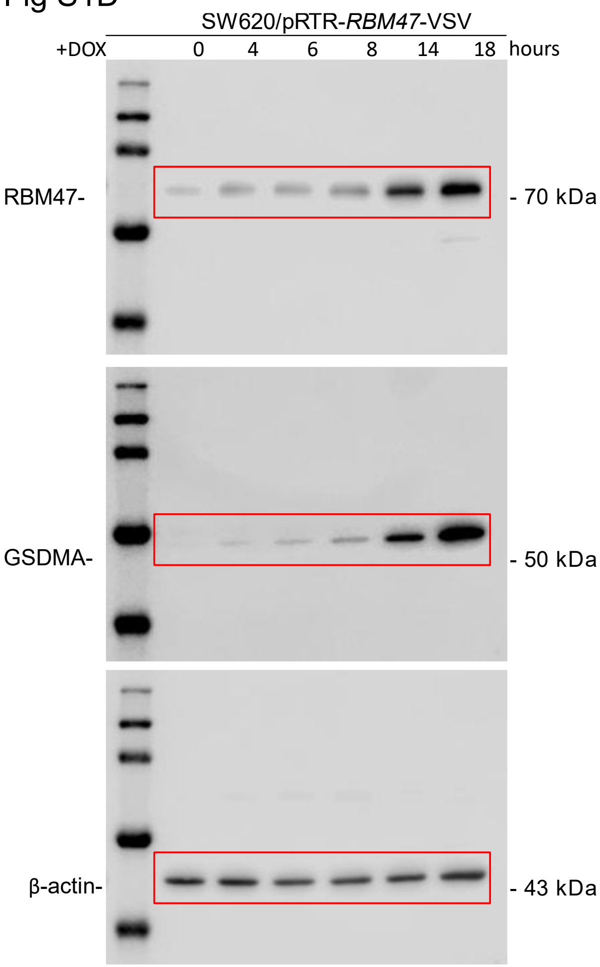

Fig 3C

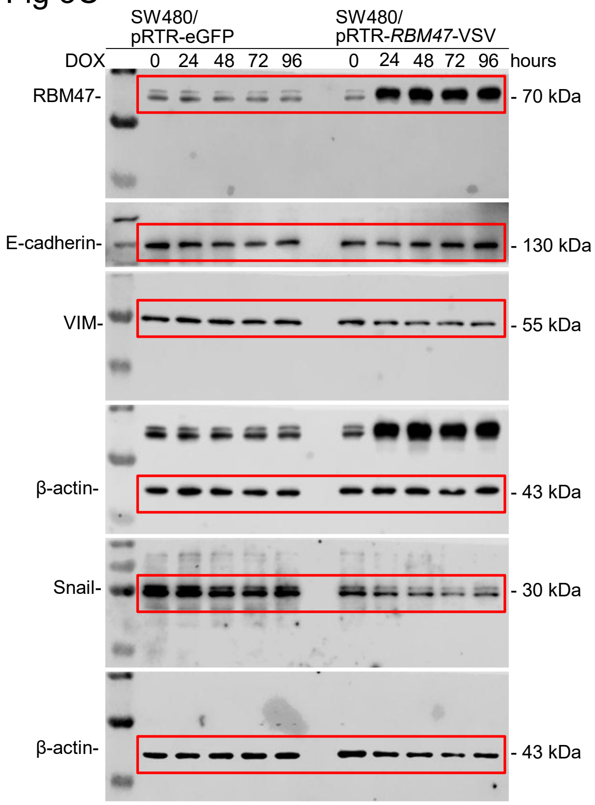

Fig S2C

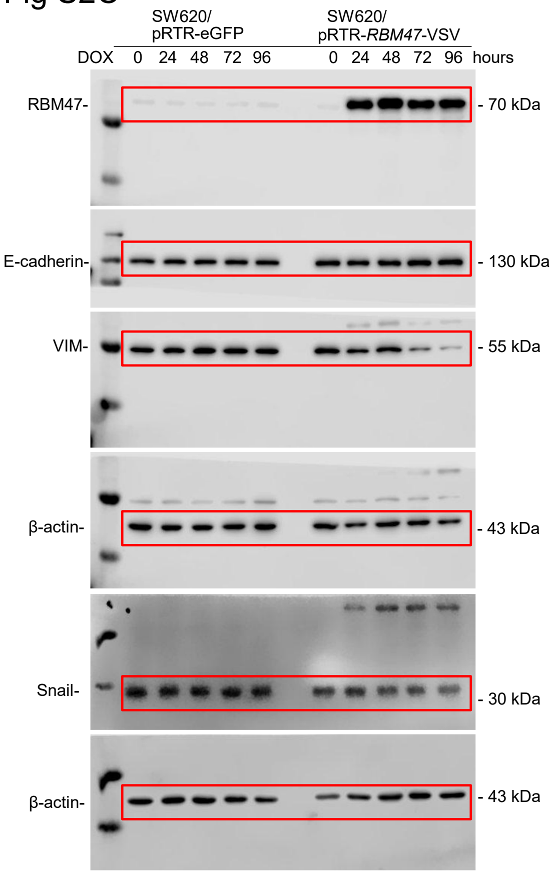

Fig 4B

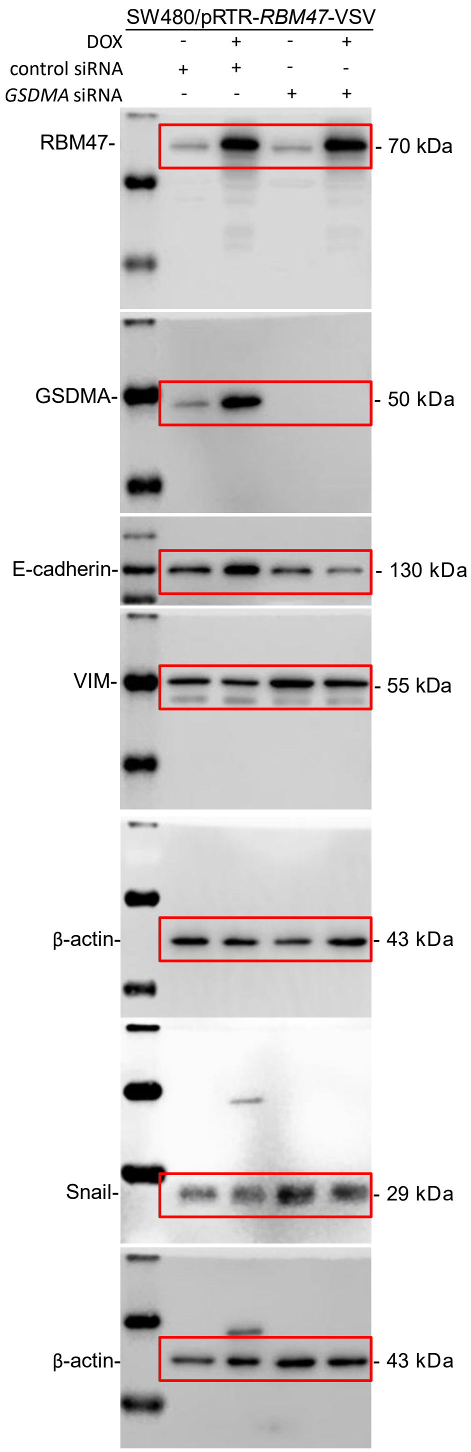

Fig S3B

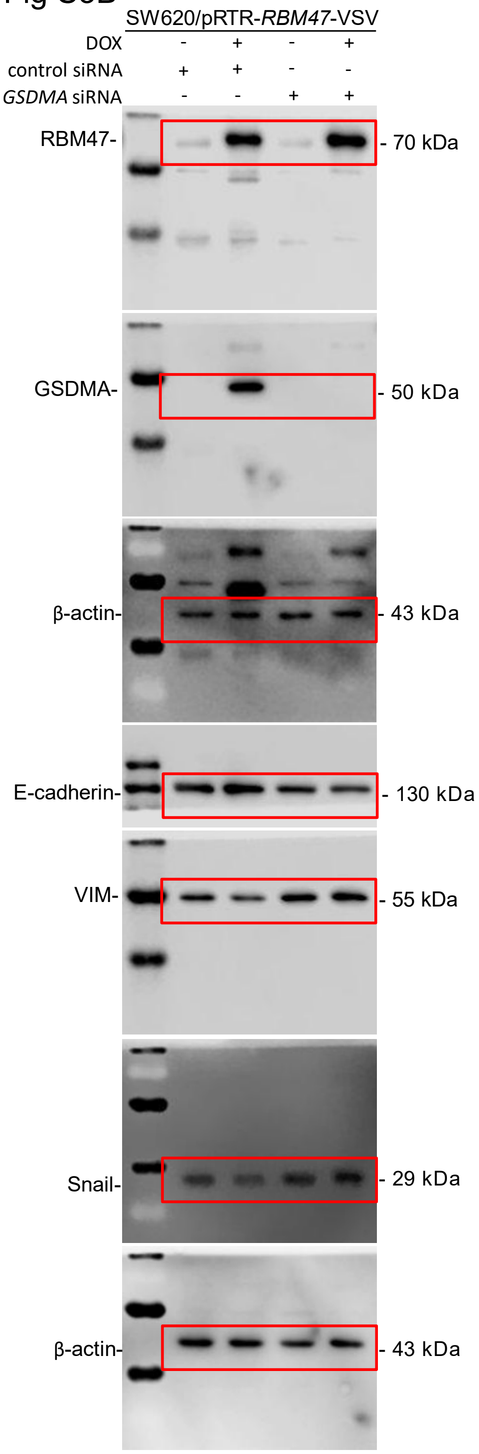

Fig 4E

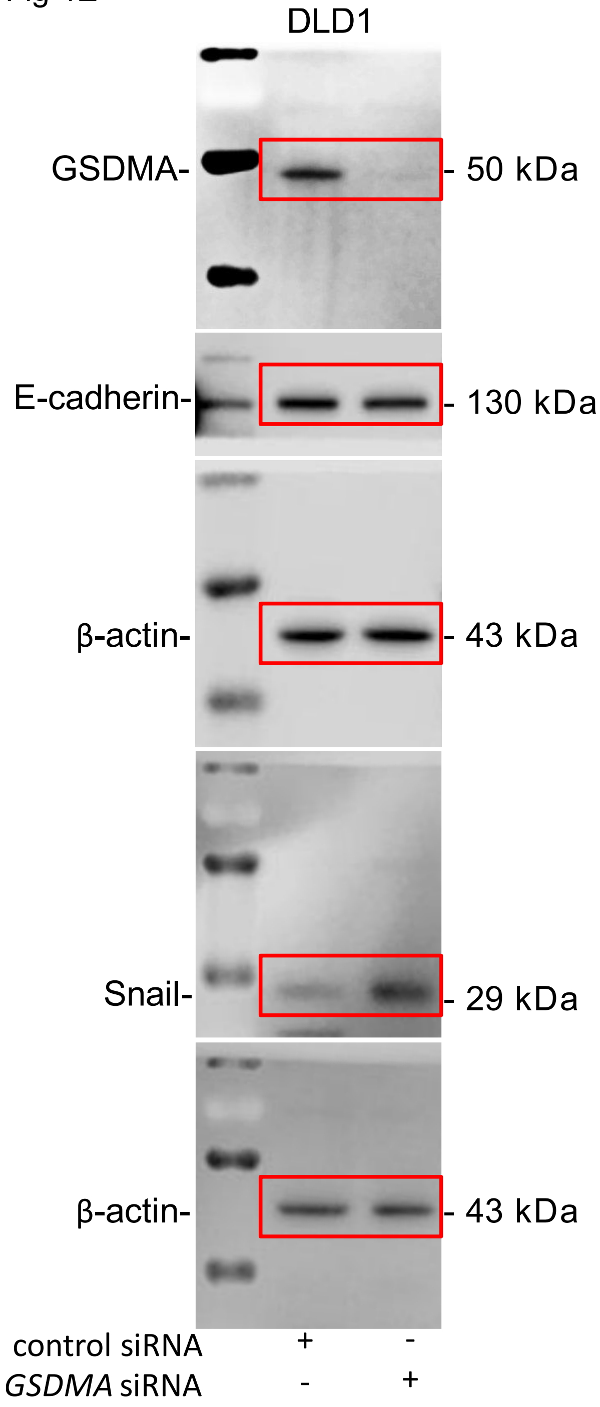

Fig S3E

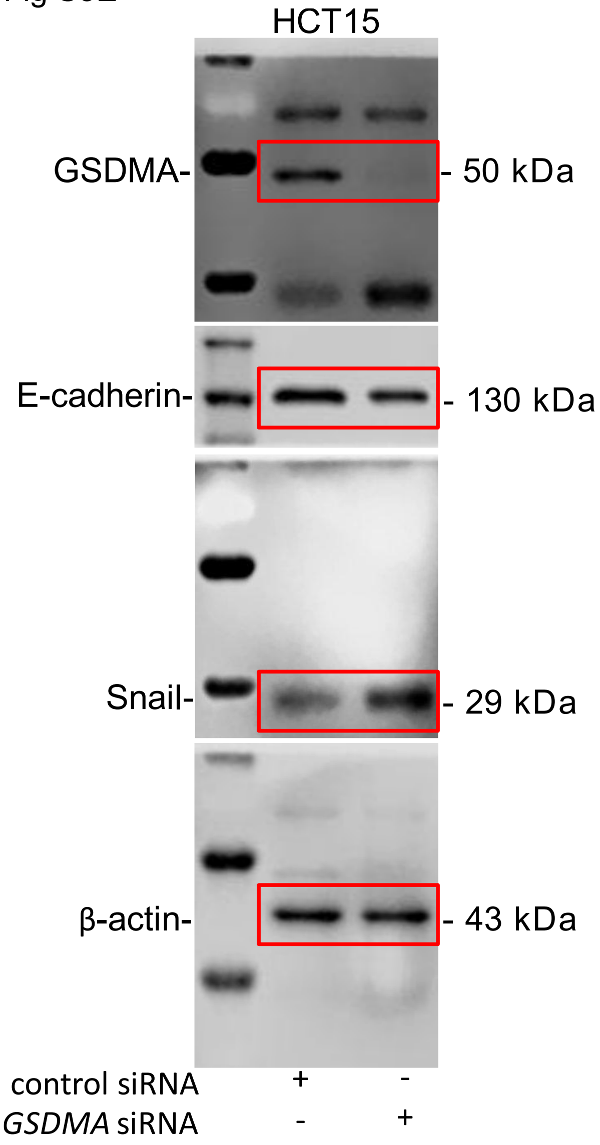

Fig 4H

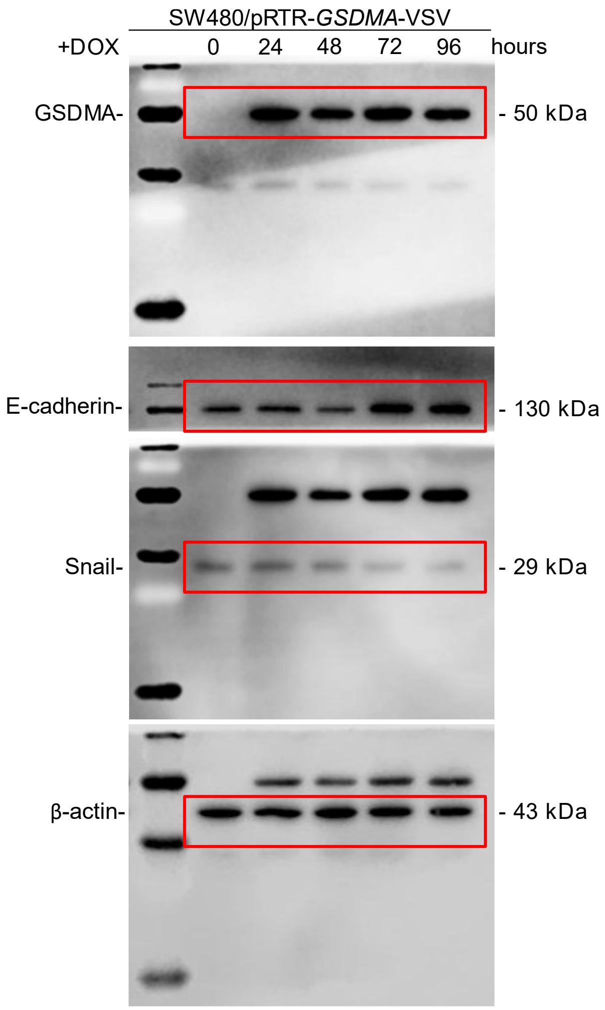

Fig S3H

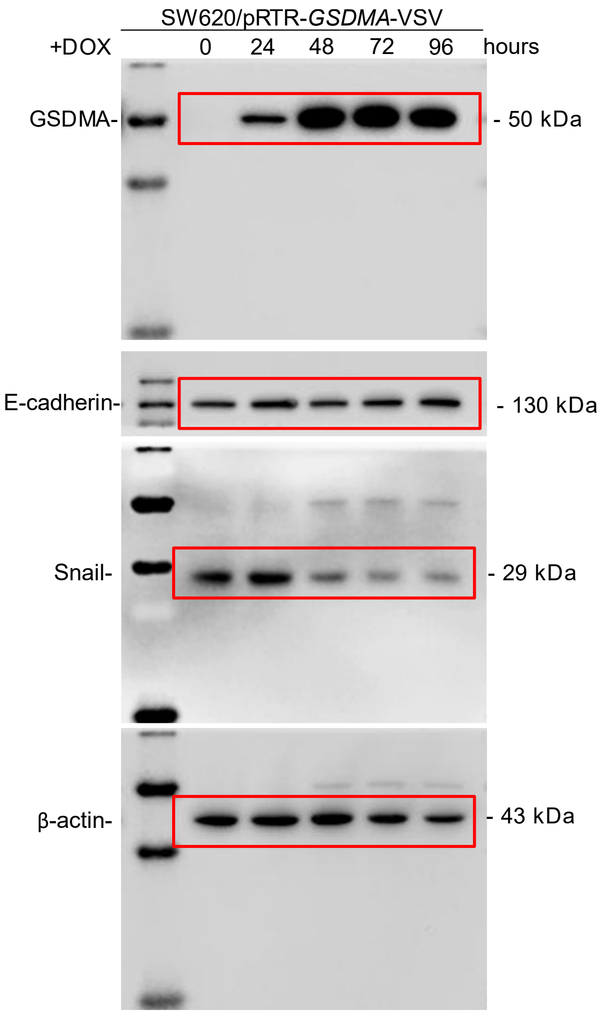

Fig S4A

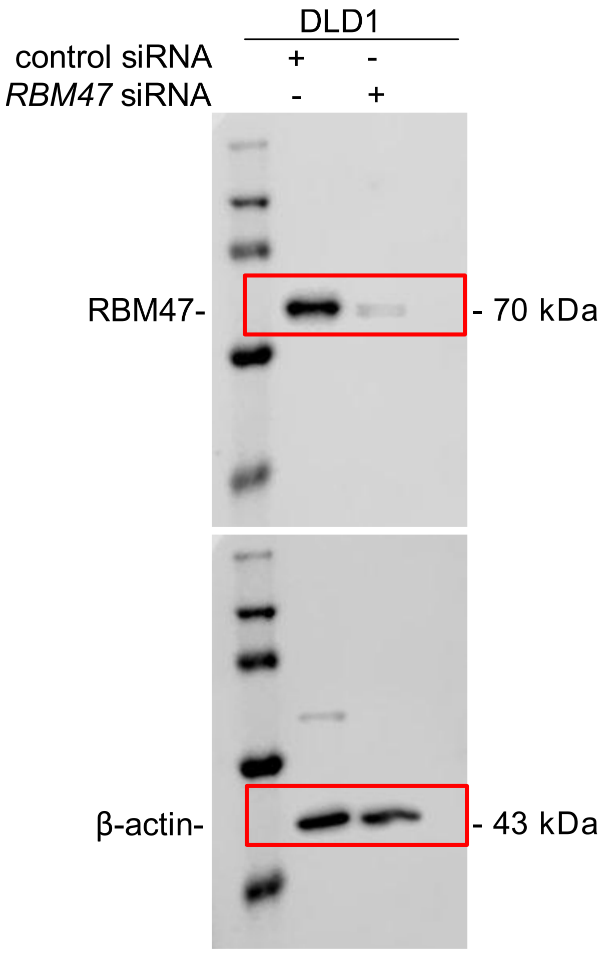

Fig S4B

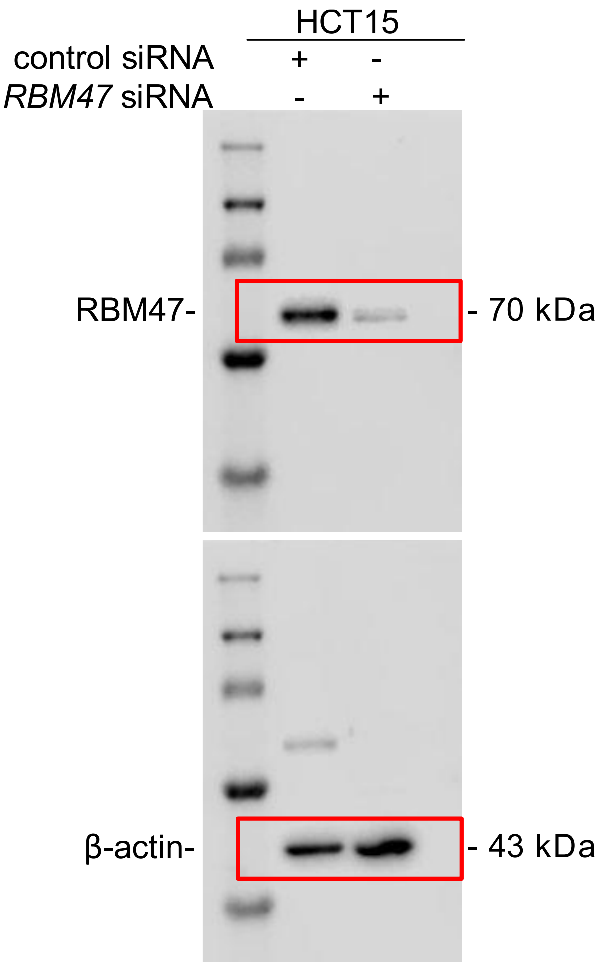

Fig S5C

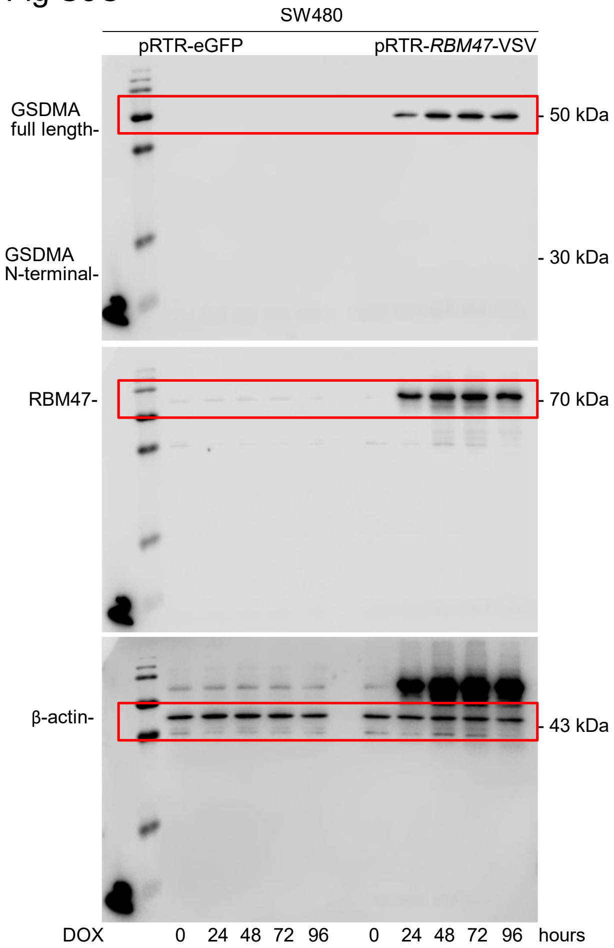

SW480
pRTR-eGFP
pRTR-*RBM47*-VSV
GSDMA full length-
GSDMA N-terminal-
RBM47-
β-actin-
50 kDa
30 kDa
70 kDa
43 kDa
DOX
0 24 48 72 96 0 24 48 72 96 hours

Fig 6C

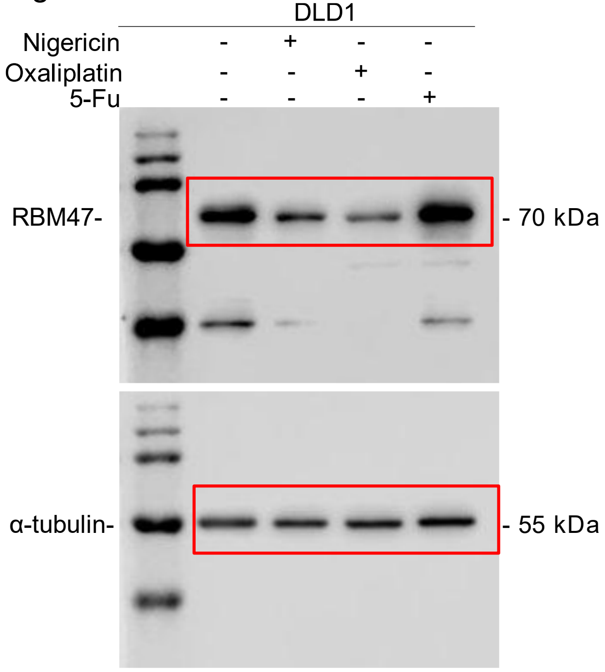

Fig S6A

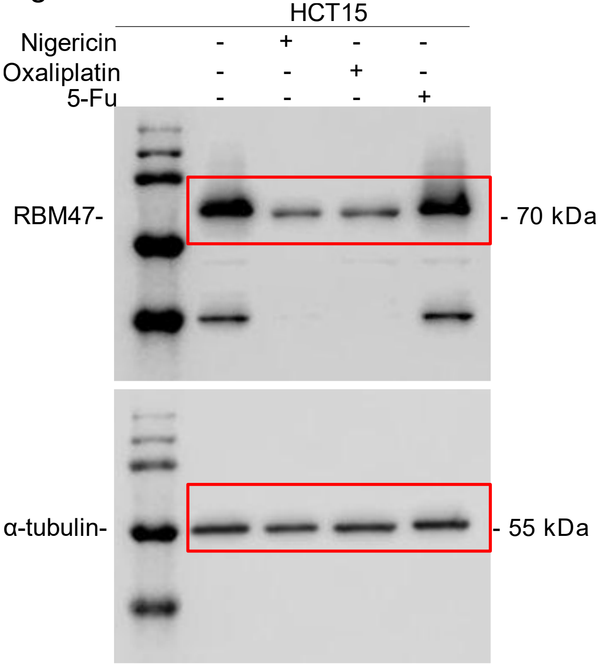

Supplement: Supplementary file 1 [file cancers-18-00504-s001.zip › cancers-4100443-supplementary.pdf]
